# OsCDC48/48E complex is required for plant survival in rice (*Oryza sativa* L.)

**DOI:** 10.1007/s11103-019-00851-9

**Published:** 2019-04-01

**Authors:** Lei Shi, Xiao-bo Zhang, Yong-feng Shi, Xia Xu, Yuqing He, Guosheng Shao, Qi-na Huang, Jian-li Wu

**Affiliations:** 1 State Key Laboratory of Rice Biology, China National Rice Research Institute, 359 Tiyuchang Road, Hangzhou, 310006 China; 2 National Key Laboratory of Crop Genetic Improvement, Huazhong Agricultural University, Wuhan, 430070 China

**Keywords:** Rice, Premature senescence and death, AAA-ATPase, Cell division cycle, CDC48

## Abstract

**Key message:**

We demonstrate that the C-terminus of OsCDC48 is essential for maintaining its full ATPase activity and OsCDC48/48E interaction is required to modulate cellular processes and plant survival in rice.

**Abstract:**

Cell division cycle 48 (CDC48) belongs to the superfamily protein of ATPases associated with diverse cellular activities (AAA). We previously isolated a rice CDC48 mutant (*psd128*) displaying premature senescence and death phenotype. Here, we showed that OsCDC48 (Os03g0151800) interacted with OsCDC48E (Os10g0442600), a homologue of OsCDC48, to control plant survival in rice. OsCDC48E knockout plants exhibited similar behavior to *psd128* with premature senescence and plant death. Removal of the C-terminus of OsCDC48 caused altered expression of cell cycle-related genes, changed the percentage of cells in G1 and G2/M phases, and abolished the interaction between OsCDC48 itself and between OsCDC48 and OsCDC48E, respectively. Furthermore, the truncated OsCDC48–PSD128 protein lacking the C-terminal 27 amino acid residues showed a decreased level of ATPase activity. Overexpression of *OsCDC48–psd128* resulted in differential expression of AAA-ATPase associated genes leading to increased total ATPase activity, accumulation of reactive oxygen species and decreased plant tiller numbers while overexpression of *OsCDC48* also resulted in differential expression of AAA-ATPase associated genes leading to increased total ATPase activity, but increased plant tiller numbers and grain yield, indicating its potential utilization for yield improvement. Our results demonstrated that the C-terminal region of OsCDC48 was essential for maintaining the full ATPase activity and OsCDC48/48E complex might function in form of heteromultimers to modulate cellular processes and plant survival in rice.

**Electronic supplementary material:**

The online version of this article (10.1007/s11103-019-00851-9) contains supplementary material, which is available to authorized users.

## Introduction

The eukaryotic *cell division cycle 48* (*cdc48*) mutant was originally isolated among a collection of cold-sensitive yeast (*Saccharomyces cerevisiae*) mutants defective in cell cycle (Moir et al. 1982). p97 (named after its molecular weight of 97 kDa), the mammalian homologue of CDC48, was identified in various cells/tissues of many species (Peters et al. 1990). To date, it has been shown that CDC48/p97 was involved in a wide range of cellular processes such as regulation of cell cycle progression, cell growth and proliferation, apoptosis and necrosis, ubiquitination protein degradation and membrane fusion processes in archeabacteria, yeasts, animals and plants (Golbik et al. 1999; Mouysset et al. 2008; Bègue et al. 2016; Huang et al. 2016).

CDC48/p97 belongs to the superfamily protein of ATPases associated with diverse cellular activities. A typical CDC48/p97 usually consists of an N-terminal domain, one or two copies of AAA domain, and a C-terminal domain (Dalal and Hanson 2001; Wang et al. 2004). Based on the number of AAA domain, the members of the AAA gene family can be classified into two types: Type I contains only a single copy of AAA domain and Type II contains two copies of AAA domain termed D1 and D2, respectively (Wang et al. 2004). The AAA domain comprises the conserved motifs (Walker A and Walker B) for ATP binding and hydrolysis (Wang et al. 2003a, b; Zhang et al. 2000). The N domain and C tail are structurally flexible and mainly involved in selecting and/or processing cofactors/substrates, but they could also modulate ATPase activity via either posttranslational modification or protein–protein interaction (Ye 2006; Jentsch and Rumpf 2007; Meyer et al. 2012). In the case of Type II family members, the conformation of the N-domain in relation to the D1-D2 hexamer is directly linked to ATP hydrolysis and that the C-terminal region is required for the hexamer stability (Niwa et al. 2012).

The physiological and biochemical functions of CDC48/p97 protein are mainly manifested by the activity of AAA-ATPase (Peters et al. 1990). It promotes the assembly and disassembly of proteins by using the ATPase activity, and ultimately achieves diverse cellular functions (Xia et al. 2016). AAA proteins exist in both monomeric and oligomeric forms, and only oligomeric form that mostly oligomerizes into hexameric, ring-like structures that act upon their substrates (Dalal and Hanson 2001; Niwa et al. 2012; Rockel et al. 2002; Rouiller et al. 2002; Zhang et al. 2000). CDC48/p97, an important member of the eukaryotic Type II AAA ATPase, is highly conserved in molecular evolution and involved in the regulation of diverse cellular activities (Ballar et al. 2011; Bègue et al. 2016; Franz et al. 2011; Mouysset et al. 2008). It has been shown that mutations in CDC48/p97 would result in a wide range of abnormal phenotypes including lethality of an organism. The substitution of S565G in CDC48 causes altered cold sensitivity and cell apoptosis in *S. cerevisiae* (Madeo et al. 1997). Overexpression of zebrafish CDC48 promotes cell proliferation and increases DNA synthesis while the expression of a mutant molecule with a tyrosine-805 to alanine substitution at the C-terminal phosphorylation site inhibits cell proliferation and induces apoptosis at low temperatures (Imamura et al. 2003). Overexpression of *Trypanosome brucei* VCP-1, a CDC48/p97 homologue with a D2 mutation, could completely terminate the cell cycle, thus leading to growth arrest and apoptosis (Lamb et al. 2001). Mutations and RNAi of CDC48 have been found to cause embryonic lethal, abnormal disassembly of spindles, insufficient DNA synthesis and endoplasmic reticulum-associated degradation of proteins in *X. laevis* and *C. elegans* (Cao et al. 2003; Mouysset et al. 2008; Ballar et al. 2011). In mammals, CDC48/p97 regulates the process of apoptosis, especially in the pathogenesis of myopathy associated with Paget’s disease of bone and frontotemporal dementia (Haubenberger et al. 2005; Guinto et al. 2007). In human, a point mutation in p97 causes retarded cell proliferation and apoptosis of human B-lymphocytes (Shirogane et al. 1999). In rice, both a single base substitution in *OsCDC48* and RNAi of *OsCDC48* result in premature senescence and plant death (Huang et al. 2016). Although the mechanism of CDC48-mediated cell death remains to be further elucidated, it is believed that the diverse CDC48 functions depend largely on its cofactors such as the serine/threonine kinase Pim-1 (Shirogane et al. 1999) and the deubiquitinating enzyme ATX-3 (Kuhlbrodt et al. 2011). Thus, identification of cofactors is crucial for the understanding of CDC48-mediated cellular processes.

The rice genome (*Oryza sativa* L. Japonica. cv. Nipponbare) harbors 29 members of AAA proteins including CDC48 (Os03g0151800). However, very little is known about the roles of OsCDC48 in rice growth, development and senescence. We previously identified a rice *premature senescence and death 128* (*psd128*) mutant resulting from a point mutation of *OsCDC48*, and an OsCDC48 homologue termed as OsCDC48E (Huang et al. 2016). In this study, we found that OsCDC48E knockout plants exhibited similar behavior to *psd128* with premature senescence and death phenotype. The interaction of OsCDC48 with OsCDC48E (Os10g0442600) was necessary for rice plant survival. Removal of the C-terminus of OsCDC48 decreased its ATPase activity, caused the altered expression of cell cycle-related genes, changed the percentage of cells in G1 and G2/M phases, and abolished the interaction between OsCDC48 itself and between OsCDC48 and OsCDC48E, respectively. Overexpression of *OsCDC48–psd128* resulted in differential expression of AAA-ATPase associated genes leading to increased total ATPase activity, accumulation of reactive oxygen species and decreased plant tiller numbers while overexpression of *OsCDC48* also resulted in differential expression of AAA-ATPase associated genes leading to increased total ATPase activity, but increased plant tiller numbers and grain yield, indicating its potential utilization for yield improvement. Our results demonstrated that the C-terminal region of OsCDC48 was essential for maintaining the full ATPase activity and OsCDC48/48E complex was likely functioning as heteromultimers to regulate cellular processes and plant survival in rice.

## Materials and methods

### Plant materials and growth conditions

The *premature senescence and death 128* (*psd128*) mutant was obtained from a mutant population generated by ethyl methane sulfonate (EMS) treatment of IR64, an elite *indica* rice (*Oryza sativa* L.) cultivar (Huang et al. 2016). The wild type IR64 (WT), *psd128* and three backcross F_3_ lines (psd128/IR64//IR64) with the mutant phenotype were planted in the paddy field under regular water, fertilizer and pest management in the summer of 2017 at the China National Rice Research Institute (CNRRI) in Fuyang, Hangzhou, China. Transgenic plants including overexpression and CRISPR/Cas9-mediated knockout plants were grown in the greenhouse at CNRRI.

### Vector construction

For overexpression analysis, the full-length complementary DNAs (cDNAs) of *OsCDC48* and *OsCDC48–psd128* were amplified, respectively, and the PCR products were then cloned into the binary vector pCambia1305.1-GFP using the Trelief™ SoSoo Cloning Kit (Tsingke, Hangzhou, China) to generate two new constructs pd35S::OsCDC48-GFP and pd35S::psd128-GFP. The pd35S::OsCDC48-GFP and pd35S::psd128-GFP constructs were introduced into the calli generated from the mature embryo of WT, respectively. To knock out *OsCDC48E*, the targeted deletion vector was constructed following the CRISPR/Cas9 system (Wang et al. 2015) using the target sequence, 5′-CAGGCCTGACATCATAGATC-3′, and introduced into the calli induced from the mature embryo of WT via *Agrobacterium tumefaciens*-mediated transformation (Hiei and Komari 2008).

### Histochemical analysis, H_2_O_2_ and malonaldehyde and pigment level determination

Young leaves from WT and overexpression plants at the tillering stage were used for 3,3′-diaminobenzidine (DAB), tryphan blue staining and H_2_O_2_ and malonaldehyde (MDA) content determination. The DAB assay was carried out according to method described by Thordal-Christensen et al. (1997). Tryphan blue staining was carried following the method described by Yin et al. (2000). The H_2_O_2_ and MDA contents were determined using the method described by Moradi and Ismail (2007). Chlorophyll (Chl) a and Chl b contents were measured using young leaves from WT and CRIPSR-Cas9 knockout plant Cr9-2# at the tillering stage according to the method described by Arnon (1949) while the carotenoid contents from the same leaves were determined following the method described by Wellburn (1994). The means from three replicates were used for analysis.

### Flow cytometric analysis

To prepare suspension cells, the 3-day-old shoots of WT and *psd128* after germination were soaked in 500 µl 2 µg/ml DAPI solution (DAPI, Beckman, NPE 731085) in a 1.5 ml Eppendorf tube on ice, then cut into pieces and chopped with a sharp razor blade. After filtering the slurry through a 40 µm nylon filter (FALCON 352340), the suspension of nuclei was loaded into a Beckman Moflo-XDP (Beckman Coulter, Inc., CA, USA) for flow cytometric analysis as described by Galbraith et al. (1983), and the ploidy of approximate 10,000 nuclei was recorded for each test. The numbers of diploid and tetraploid nuclei were recorded, and the relative proportions of G1, S, and G2/M cells were calculated using the Summit 2.0 software (Beckman Coulter, USA).

### Gene expression analysis

Total RNA was extracted from various organs (flag leaf, flag leaf sheath, culm, root, panicle) of WT and *psd128* using Trizol reagent (Invitrogen, Life Technologies, Carlsbad, CA, USA) following the manufacturer’s protocol. The first-strand cDNA was synthesized from DNaseI-treated RNA with an oligo (dT)_18_ primer in a 20 µL reaction using the ReverTra Ace qPCR Master Mix kit (Toyobo, Tokyo, Japan) following the manufacturer’s protocol. qRT-PCR was conducted using SYBR® Premix Ex TaqTM II (Tli RNaseH Plus) Kit and performed on a Thermal Cycle Dice® Real Time System (TaKaRa, Dalian, China). The rice ubiquitin gene *Os03g0234200* was used as an internal control. For spatial and temporal expression analysis, total RNA samples were prepared from the flag leaves, flag leaf sheaths, culms, roots, and panicles of WT and *psd128* at the heading stage. For ATPase-related gene expression analysis, total RNA samples were prepared from 15 day-old seedlings of WT and *psd128*. For cell cycle-related gene expression analysis, total RNA samples were prepared from 3 day-old shoots. In addition, total RNA samples were prepared for CDC48/48E expression analysis from the leaves of CRISPR/Cas9 knockout plants at the tillering stage. All primers used for qRT-PCR are listed in Supplementary Table S1. All assays were repeated at least three times, and the means were used for analysis. The 2^− ΔΔCt^ method (Schmittgen and Livak 2008) was used to calculate relative transcript abundances.

### Transcriptome analysis

To eliminate other possible mutations in the mutant, *psd128* was first backcrossed to WT. The F_1_ plants were then backcrossed to WT again to generate BC_2_F_1_, which was selfed to generate BC_2_F_2_ and BC_2_F_3_. Three biological repeats with three siblings/repeat of BC_2_F_3_ were used for total RNA extraction with TRIzol reagent (Invitrogen). High throughput RNA sequencing (RNA) was performed by Novogene. Library construction was performed according to Illumina instructions and sequenced on a HiSeq 2000 sequencer. All paired-end reads were mapped to the rice cv. Nipponbare genome using TopHat2 (Trapnell et al. 2009). Expression levels were calculated using the reads per kb permillion reads method (Mortazavi et al. 2008). The DESeq R package (1.10.1) was used for analysis of differentially expression genes (DEGs) between WT and BC_2_F_3_. The DEGs were filtered for a corrected *P* ≤ 0.005. The clustered genes were assigned to biological process categories based on GO analysis using the Web tool DAVID Bioinformatics Resources 6.7 (http://david.abcc.ncifcrf.gov/home.jsp). Significantly enriched GO terms for the DEGs compared with the genomic background were identified using a hypergeometric test (*P* ≤ 0.05). KEGG pathway-based analysis was performed using the blastall program against the KEGG database (http://www.genome.jp/kegg). Significantly enriched metabolic pathways or signal transduction pathways for the DEGs were identified by pathway enrichment analysis (*P* ≤ 0.05; Kanehisa et al. 2008).

### Subcellular localization

The full-length CDSs of *OsCDC48, OsCDC48E* and *OsCDC48–PSD128* were amplified and cloned into PAN580 to generate three transient expression vectors p35S-CDC48-GFP, and p35S-CDC48E-GFP, p35S-CDC48-PSD128-GFP, respectively. The nuclear marker mCherry-D53 and the cytoplasmic marker mCherry-TAD1 were amplified and cloned into 163-mCherry (Xu et al. 2012; Zhou et al. 2013). The vectors p35S-CDC48-GFP and p35S-CDC48E-GFP were respectively co-transformed with mCherry-D53 and mCherry-TAD1 into the rice protoplasts which were prepared from the stem and leaf sheath of 15-day-old rice seedlings according to the method described by Chen et al. (2006). In addition, p35S-CDC48-GFP and p35S-CDC48-PSD128-GFP were introduced into the protoplasts using the same method, respectively (Chen et al. 2006).The GFP fluorescence was observed by a Zeiss Ism710 confocal laser scanning microscope (Carl Zeiss, Inc., Jena, Germany).

### Protein purification and ATPase activity assay

For the recombinant protein expression in *Escherichia coli*, the CDS of *OsCDC48*, OsCDC48–*PSD128* and *OsCDC48E* were amplified and cloned into pET28a (Merck, Darmstadt, Germany). In addition, the CDS of *OsCDC48* and OsCDC48–PSD*128* were also cloned into pGEX-4T-1 (GE Healthcare, Chicago, USA). Recombinant proteins were induced in *E. coli* strain Rossetta, and purified by Glutathione-Sepharose Resin Protein Purification Kit and 6 × His-Tagged Protein Purification Kit (CWBIO, Beijing, China) according to the manufacture’s instruction, respectively. Both fresh leaf tissues from 15 day-old seedlings of WT, *psd128* and overexpression transgenic T_1_ lines and recombinant protein (His-tag) were used to measure ATPase activity by the ATPase Test Kit according to the manufacture’s instruction (Jiancheng Bioengineering Institute, Nanjing, China).

### Western blot assay

For immunoblot analysis, the recombinant proteins (His-tag) were separated on 10% SDS–PAGE gel. Then the target protein bands were sequentially detected with primary antibody anti-His (Cat: CW0083, CWBIO, Beijing, China, 1:5000 dilution) at 4 °C overnight, and secondary antibody with a HRP (Cat: 33101ES60, YEASEN, Shanghai, China, 1:1000 dilution) for 1 h, the immunoblot signal was detected using Supersignal West Pico Chemiluminescent Substrate (Thermo, Waltham, USA) and visualized by the ChemDocTM Touch Imaging system (Bio-Rad, CA, USA).

### Yeast two-hybrid and in vitro pull-down assay

Yeast two-hybrid assays were performed with the Y2H Gold-Gal4 system (Clontech, http://www.clontech.com). DNA fragments containing the full coding sequences of *OsCDC48, OsCDC48E* and *OsCDC48–psd128* genes were inserted into the pGBKT7 and pGADT7 vectors to from the bait and prey constructs, respectively. The bait and prey constructs were transformed into yeast strain Y2H Gold according to the manufacturer’s instruction (Clontech, http://www.clontech.com). The yeast cells were cultured on SD/-Trp-Leu or SD/-Trp-Leu-His-Ade medium containing X-ɑ-gal and Aureobasidin A (AbA) at 30 °C in darkness for 3 days. The pull-down assay was conducted as follows: 50 µL equilibrated Glutathione High Capacity Magnetic Agarose Beads (Sigma, St Louis, USA) was mixed with 500 µg of each recombinant protein in 600 µL pull-down buffer (50 Mm Tris–HCl pH = 7.5, 5% glycerol, 1 mM EDTA, 1 mM DTT, 1 Mm PMSF, 0.01% Nonidet P-40, 150 mM KCl) under 4 °C for 2 h. The bound proteins together with the beads were collected by a magnetic shelf (Invitrogen, Carlsbad, USA), washed with pull-down buffer twice, eluted with 50 µL 1 × PBS and immune detected by GST (Cat: CW0085, CWBIO, Beijing, China), HIS (Cat: CW0083, CWBIO, Beijing, China) antibodies respectively.

### BiFC assay

For the BiFC assay, the full length CDSs of *OsCDC48, OsCDC48E*, and *OsCDC48–psd128* genes were cloned into pCAMBIA1300S-YN and pCAMBIA2300S-YC to form the nYFP-protein and cYFP-protein constructs, respectively. The constructs then were transformed into rice protoplasts according to the protocols described previously by Zhang et al. (2011). In addition, the constructs also were transiently expressed in tobacco leaves following the method described by Waadt and Kudla (2008). The BiFC assay was performed as described previously (Waadt and Kudla 2008). A confocal laser scanning microscope (Zeiss LSM710) was used to detect YFP fluorescent signals after 48 h post-transfection.

## Results

### OsCDC48E belongs to the AAA-ATPase family protein

We previously isolated *OsCDC48* in a study of the rice *premature senescence and death* 128 (*psd128*) mutant (Huang et al. 2016). A single base substitution at position C2347T leading to a premature stop mutation (*OsCDC48*–*PSD128*) resulting in a putative truncated protein (OsCDC48–PSD128) lacking of 27 amino acid residues at the C-terminus, is responsible for the premature senescence and death phenotype of *psd128*. We also identified an unknown function OsCDC48 homologue, OsCDC48E, which is localized to chromosome 10 and shares 97% identity to OsCDC48 at the amino acid level (Huang et al. 2016). In the present study, we showed that the cDNA of *OsCDC48E* (accession number, MK292711) compromised of 2882 bp including a 145 bp 5′-UTR, a 310 bp 3′-UTR and a 2427 bp CDS. Similar to *OsCDC48, OsCDC48E* has 9 exons and 8 introns, and encodes a putative AAA-ATPase with 808 amino acid (aa) residues with a predicted molecular mass of 96.96 kDa. Similar to OsCDC48, the putative OsCDC48E protein has a typical N terminus (1–199 aa), a C terminus (772–808 aa) and two AAA-ATPase domains (D1 from 213 to 466 aa and D2 from 486 to 771 aa) (Fig. S1). Each of the AAA-ATPase domains contains the Walker A and Walker B motifs (Fig. S1). The results indicated that OsCDC48E belongs to the AAA-ATPase family protein.

### *OsCDC48E* is constitutively expressed

**Fig. 1:**
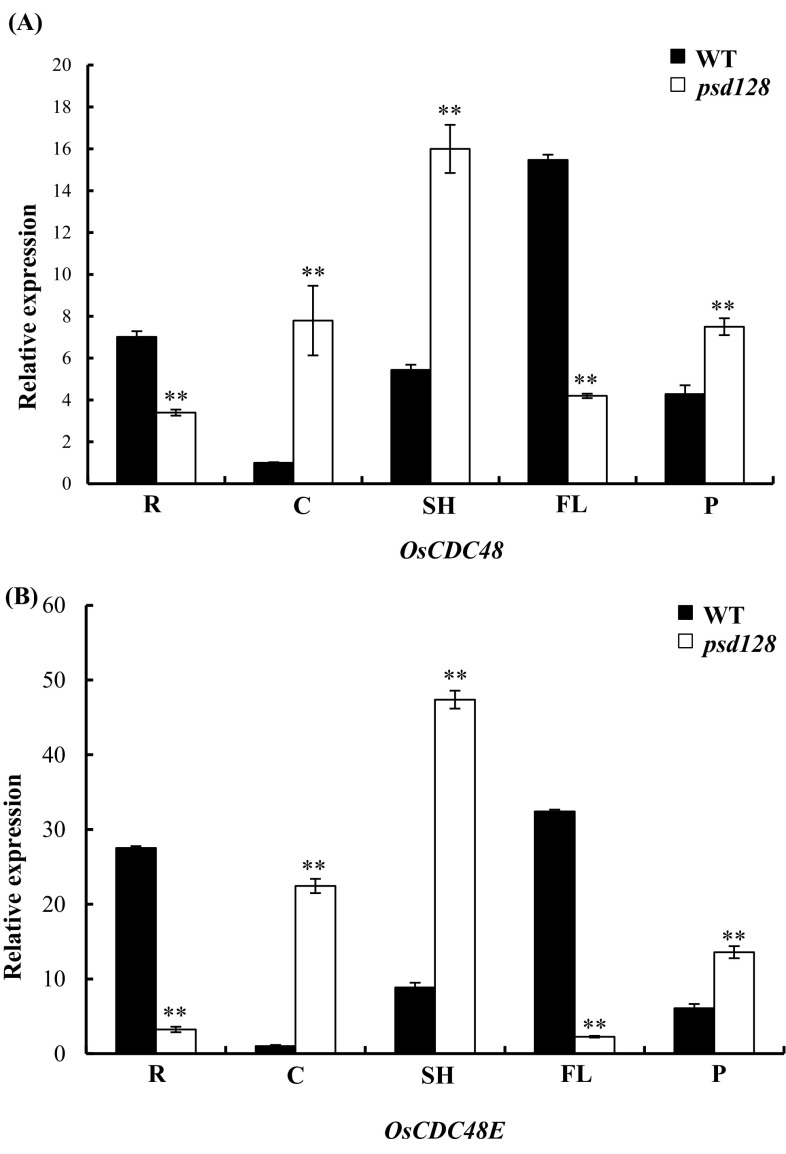
Expression of *OsCDC48* and *OsCDC48E* in differrent tissues. *OsCDC48* (**a**) and *OsCDC48E* (**b**) expression in roots (R), culms (C), shoots (SH), flag leaves (FL) and panicles (P) at the heading stage. Values are means ± *SD* from three biological replicates. Significance at **P ≤ 0.01 by Student’s *t*-test

*OsCDC48* is constitutively expressed in the tissues tested (Huang et al. 2016). To determine the expression pattern of *OsCDC48E*, Real-time PCR was carried out and the results revealed that *OsCDC48E* had a similar expression pattern to *OsCDC48*, and was constitutively expressed in different tissues including the roots, culms of the second internode, flag leaf sheaths, flag leaves and panicles at the heading stage (Fig. 1). The highest expression levels of *OsCDC48* and *OsCDC48E* were detected in the flag leaves while the lowest levels of *OsCDC48* and *OsCDC48E* were detected in the culms in IR64 (WT). In contrast, the highest expression levels of *OsCDC48* and *OsCDC48E* were detected in the flag leaf sheaths while the lowest levels of *OsCDC48* and *OsCDC48E* were detected in the roots and flag leaves in *psd128* (Fig. 1). These results suggested that the expression patterns of *OsCDC48* and *OsCDC48E* were similar both in *psd128* and WT at the heading stage.

### OsCDC48E and OsCDC48 localize to nucleus and cytoplasm

**Fig. 2:**
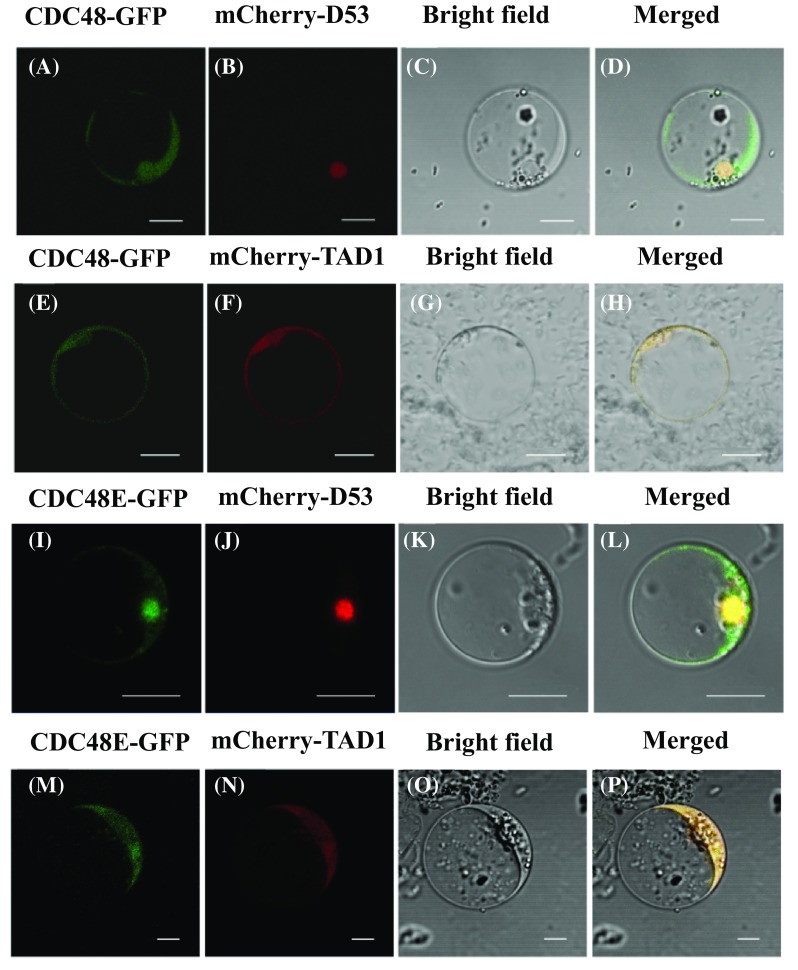
Subcellular localization of OsCDC48 and OsCDC48E in rice protoplast. **a**–**d** Subcellular localization of OsCDC48-GFP. The nuclear marker OsD53 fused with mCherry was used as a positive control. Bar = 10 µm; **e**–**h** subcellular localization of OsCDC48-GFP. The cytoplasmic marker OsTAD1 fused with mCherry was used as a positive control. Bar = 10 µm; **i**–**l** subcellular localization of OsCDC48E-GFP. The nuclear marker OsD53 fused with mCherry was used as a positive control. Bar = 10 µm; **m**–**p** subcellular localization of CDC48E-GFP. The cytoplasmic marker OsTAD1 fused with mCherry was used as a positive control. Bar = 5 µm

In the previous study, we localized OsCDC48-GFP to the nucleus and cytoplasm in the mesophyll cells of *Nicotiana tabacum* (Huang et al. 2016). To confirm the subcellular localization of OsCDC48-GFP in rice nuclei, the transient vector p35S-CDC48-GFP was co-transformed with the nuclear marker mCherry-D53 using rice protoplasts. The results showed that both p35S-CDC48-GFP and mCherry-D53 localized to the rice nucleus, indicating that OsCDC48-GFP was indeed localized to the nucleus (Fig. 2a–d). To confirm the subcellular localization of OsCDC48-GFP in the cytoplasm in rice, the transient vector p35S-CDC48-GFP was co-transformed with the cytoplasmic marker mCherry-TAD1. The results showed that both p35S-CDC48-GFP and mCherry-TAD1 localized to the rice cytoplasm, indicating that OsCDC48-GFP was indeed localized to the cytoplasm as well (Fig. 2e–h). To determine the subcellular location of OsCDC48E in rice, similar co-transformation assays with mCherry-D53 and mCherry-TAD1 were carried out using rice protoplasts, and the results showed that OsCDC48E localized both to the nucleus (Fig. 2i–l) and cytoplasm (Fig. 2m–p). To determine the effect of C-terminal deletion on subcellular location, the transient expression vector pCDC48-PSD128-GFP was introduced into rice protoplasts, and the results showed that OsCDC48–PSD128-GFP localized to the nucleus and cytoplasm similar to OsCDC48-GFP and OsCDC48E-GFP (Fig. S2), indicating that the deletion of 27 aa residues at the C-terminus did not affect the subcellular location of OsCDC48. Taken together, our results confirmed that both OsCDC48 and OsCDC48E localized to the nucleus and cytoplasm.

### C-terminus of OsCDC48 is essential for its ATPase activity

**Fig. 3:**
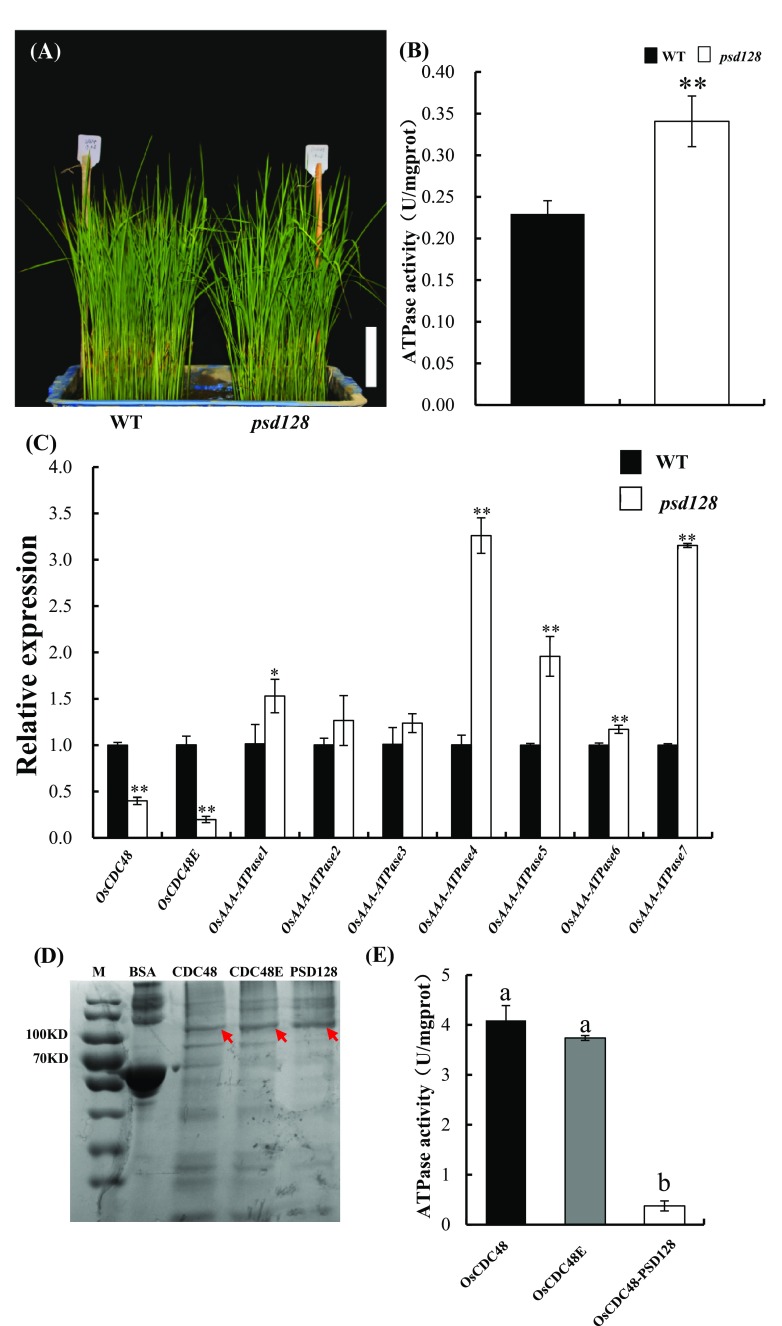
ATPase activity and expression of ATPase-related genes. **a** Two week-old seedlings of WT and *psd128* were used for analysis. Bar = 4.5 cm; **b** total ATPase activities of WT and *psd128*. Values are means ± *SD* from three biological replicates. Significance at **P ≤ 0.01 by Student’s *t*-test; **c** expression of AAA-ATPase related genes. Values are means ± *SD* from three biological replicates. Asterisks indicate significance by Student’s *t-*test (*P ≤ 0.05; **P ≤ 0.01). *OsCDC48* (Os03g0151800), *OsCDC48E* (Os10g0442600), *OsAAA-ATPase1* (Os01g0297200), *OsAAA-ATPase2* (Os07g0192800), *OsAAA-ATPase3* (Os07g0517800), *OsAAA-ATPase4* (Os12g0431100), *OsAAA-ATPase5* (Os12g0468000), *OsAAA-ATPase6* (Os12g0639200) and *OsAAA-ATPase7* (Os05g0588850); **d** purification of OsCDC48, OsCDC48E and OsCDC48–PSD128 (red arrows indicate the protein bands). The purified fraction (1 µg) was run on 10% SDS–PAGE and visualized by CBB staining. M, Protein Marker; BSA, Protein standard (20 µg); **e** ATPase activities of the purified OsCDC48, OsCDC48E and OsCDC48–PSD128, respectively, Values are means ± SD from three biological replicates. Different letter indicates significance at *P* ≤ 0.01 by Student’s *t*-test

The diverse cellular functions of CDC48/p97 protein are mainly manifested by the activity of AAA-ATPase (Peters et al. 1990). To determine whether OsCDC48 and OsCDC48E possess the ATPase activity, we first measured the total ATPase activity in 2 week-old seedling leaves of WT and *psd128* (Fig. 3a), and the results indicated that the total ATPase activity in *psd128* was significantly higher than that of WT (Fig. 3b). The results implied that other members of AAA-ATPases might contribute to the increased level of total ATPase activity in *psd128*. Therefore, we selected seven ATPase-related genes and determined their expression levels by qRT-PCR analysis. As expected, the expression levels of *OsAAA-ATPase1, OsAAA-ATPase4, OsAAA-ATPase5 OsAAA-ATPase6* and *OsAAA-ATPase7* in *psd12*8 were significantly higher than those of WT whereas the expression levels of *OsAAA-ATPase2* and *OsAAA-ATPase3* in the mutant were similar to those of WT (Fig. 3c). The results clearly demonstrated that the mutation of *OsCDC48* induced higher expression levels of a number of other AAA-ATPase genes probably for a compensation of OsCDC48 enzymatic activity. To measure the enzymatic activity of OsCDC48, OsCDC48–PSD128 as well as OsCDC48E, the corresponding proteins encoded by *OsCDC48, OsCDC48E* and *psd128* were expressed in the prokaryotic expression system, purified and identified using anti-6 × His antibody by western blot (Fig. S3), and used to measure their ATPase activities. The result showed that OsCDC48, OsCDC48E and OsCDC84-PSD128 all possessed ATPase activity. However, the activity of OsCDC48–PSD128 was significantly lower than that of the wild-type OsCDC48 (Fig. 3d, e) while the activity of OsCDC48E was similar to that of OsCDC48 (Fig. 3d, e). All these results demonstrated that OsCDC48, OsCDC48E and OsCDC48–PSD128 all possessed ATPase activity and the C-terminus of OsCDC48 was essential for the full ATPase activity of OsCDC48.

### Overexpression of *OsCDC48* increases total ATPase activity and promotes plant development

**Fig. 4:**
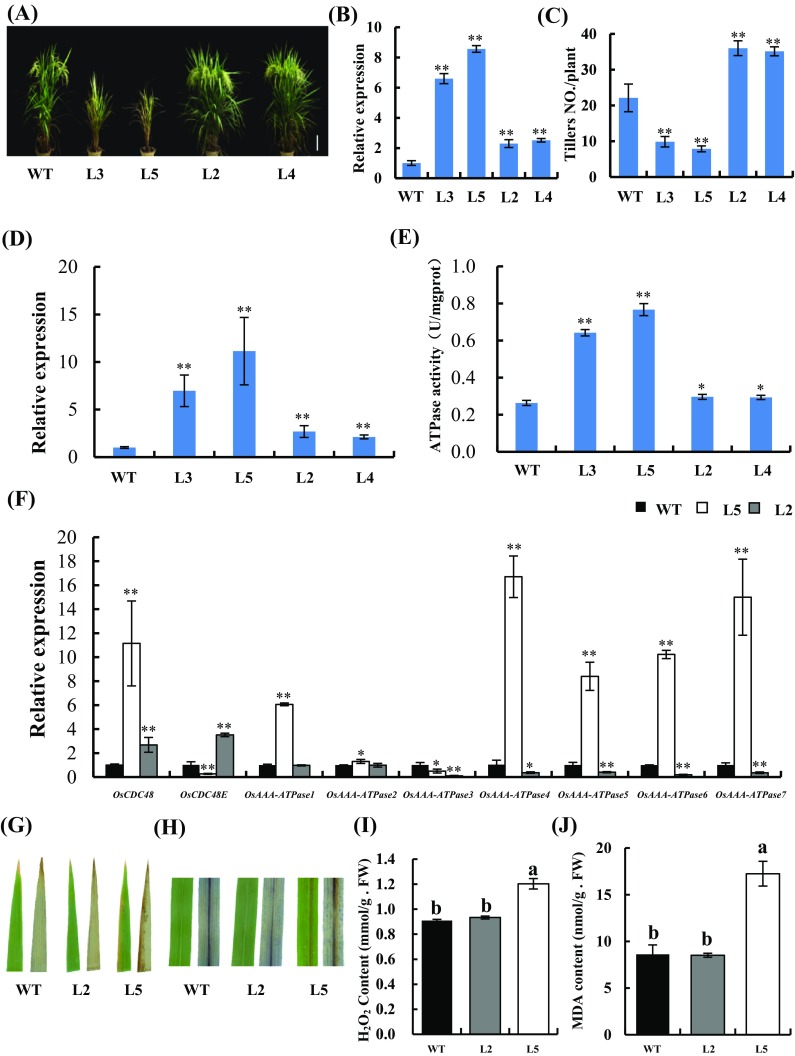
Comparison of *OsCDC48* and *OsCDC48–psd128* overexpression transgenic lines. **a** Phenotype of WT, overexpression T_0_ lines carrying pd35s::OsCDC48-GFP (L2 and L4) and pd35s::psd128-GFP (L3 and L5) at the heading stage. Bar = 20 cm; **b** relative expression of *OsCDC4*8 in overexpression T_0_ lines at the heading stage. Values are means ± SD from three biological replicates. Significance at **P ≤ 0.01 by Student’s *t*-test; **c** tiller number of T_0_ lines. Significance at **P ≤ 0.01 by Student’s *t*-test; **d** relative expression of *OsCDC4*8 in overexpression T_1_ lines of 15-day-old seedlings. Values are means ± SD from three biological replicates. Significance at **P ≤ 0.01 by Student’s *t*-test; **e** total ATPase activity of overexpression T_1_ lines of 15-day-old seedlings. Significance at **P ≤ 0.01 by Student’s *t*-test; **f** expression of AAA-ATPase related genes in overexpression T_1_ lines of 15-day-old seedlings. Values are means ± *SD* from three biological replicates. Asterisks indicate significance by Student’s *t-*test (*P ≤ 0.05; **P ≤ 0.01). *OsCDC48* (Os03g0151800), *OsCDC48E* (Os10g0442600), *OsAAA-ATPase1* (Os01g0297200), *OsAAA-ATPase2* (Os07g0192800), *OsAAA-ATPase3* (Os07g0517800), *OsAAA-ATPase4* (Os12g0431100), *OsAAA-ATPase5* (Os12g0468000), *OsAAA-ATPase6* (Os12g0639200) and *OsAAA-ATPase7* (Os05g0588850); **g** DAB staining of WT, L2 and L5 T_0_ leaves at the heading stage (Left, before staining; Right, after staining); **h** Trypan blue staining of WT, L2 and L5 T_0_ leaves at the heading stage (Left, before staining; right, after staining); **i** H_2_O_2_ contents of WT, L2 and L5 T_0_ plants at the heading stage; **j** MDA contents of WT, L2 and L5 T_0_ plants at the heading stage. Data are the means ± SD from three replicates. Different letters on the error bars indicate significance at *P* ≤ 0.01 by Duncan’s test. *FW* fresh weight

To investigate the biological effects of OsCDC48, we generated transgenic plants overexpressing the wild-type *OsCDC48* and the mutant (*OsCDC48–psd128*) alleles under the control of double 35S promoters in WT background. A total of 6 *OsCDC48* and 7 *OsCDC48–psd128* T_0_ overexpression plants were obtained respectively. Two independent plants each representing the WT (L2 and L4) and mutant type (L3 and L5) alleles were chosen for further study respectively (Fig. 4a). We found that the expression levels of *OsCDC48* and *OsCDC48–psd128* in the T_0_ plants were approximately 2- and 8- fold higher than those of WT at the heading stage, respectively (Fig. 4b). Notably, the transgenic plants overexpressing *OsCDC4*8 showed an exaggerated phenotype with significantly decreased 1000-grain weight, increased tiller number and grain yield compared with WT (Fig. 4a, c; Table S2). In contrast, the transgenic plants overexpressing *OsCDC48–psd128* showed a minimized phenotype with significantly reduced plant height, decreased tiller numbers and premature senescence similar to *psd128* compared to WT (Fig. 4a, c). The differential phenotypic performances of these transgenic plants were correlated with the expression level of the transgenes (Fig. 4b, c). Moreover, we found that the expression levels of *OsCDC48* and *OsCDC48–psd128* in the respective transgenic T_1_ lines were 2.7 (L2), 2.1(L4), 6.9 (L3) and 11.1 (L5) fold higher than those of WT at the seedling stage, respectively (Fig. 4d). Notably, all the T_1_ lines overexpressing both *OsCDC48–psd128* and *OsCDC48* at the seedling stage showed significantly increased total ATPase activity compared with WT (Fig. 4e). The results implied that other members of AAA-ATPases might also contribute to the increased level of total ATPase activity in overexpression T_1_ lines. Therefore, we further determined their expression levels of 7 ATPase-related genes in L5 (OX-OsCDC48–PSD128) and L2 (OX-OsCDC48) by qRT-PCR, respectively. The results showed that the expression levels of *OsAAA-ATPase1, OsAAA-ATPase2, OsAAA-ATPase4, OsAAA-ATPase5 OsAAA-ATPase6* and *OsAAA-ATPase7* in L5 were significantly higher than those of WT whereas the expression levels of *OsCDC48E* and *OsAAA-ATPase3* in L5 were significantly lower than those of WT (Fig. 4f). In contrast, the expression levels of *OsAAA-ATPase3, OsAAA-ATPase4, OsAAA-ATPase5, OsAAA-ATPase6* and *OsAAA-ATPase7* in L2 were significantly lower than those of WT whereas the expression levels of *OsCDC48* and *OsCDC48E* in L2 were significantly higher than those of WT, and the expression levels of *OsAAA-ATPase1, OsAAA-ATPase2* were similar to those of WT (Fig. 4f). These results demonstrated that increased total ATPase activity was contributed by other members of ATPase-related genes in L5, while increased total ATPase activity in L2 was contributed by OsCDC48 and OsCDC48E. Excessive amount of ROS causes premature senescence and cell injury/death (Davletova et al. 2005). To investigate the role of *OsCDC48* in leaf senescence, we carried out DAB and tryphan blue staining on two transgenic T_0_ plants overexpressing *OsCDC48–PSD128* (L5) and *OsCDC48* (L2) at the heading stage. The results showed that little brown precipitate and blue stains were detected on the leaves of L2, however, an apparent increased amount of brown precipitate and blue stains were detected on the leaves of L5, indicating that overexpression of the mutation allele induced H_2_O_2_ accumulation and lead to cell death (Fig. 4g, h). We then further measured the concentrations of H_2_O_2_ and MDA, the results exhibited that concentrations of both H_2_O_2_ and MDA were significantly higher in L5 leaves than those of L2 (Fig. 4i, j). Again, the severity of ROS accumulation and leaf senescence phenotype in the transgenic plants were correlated with the expression levels of the transgenes. To further explore the potential mechanism associated with premature leaf senescence in L5, we measured the expression of two senescence indicators. The results showed that the expression levels of *Osh36* and *OsI57* were apparently upregulated in L5 as well as in L3 while their expression levels were significantly downregulated in L2 as well as in L4 (Fig. S4A, B). Taken together, our results demonstrated that overexpression of *OsCDC48–psd128* induced ROS accumulation, premature leaf senescence and reduced plant tiller numbers and overexpression of *OsCDC48* delayed senescence and increased the grain yield mainly by producing more effective plant tillers.

### C terminal mutation of OsCDC48 affects cell cycle progression

**Fig. 5:**
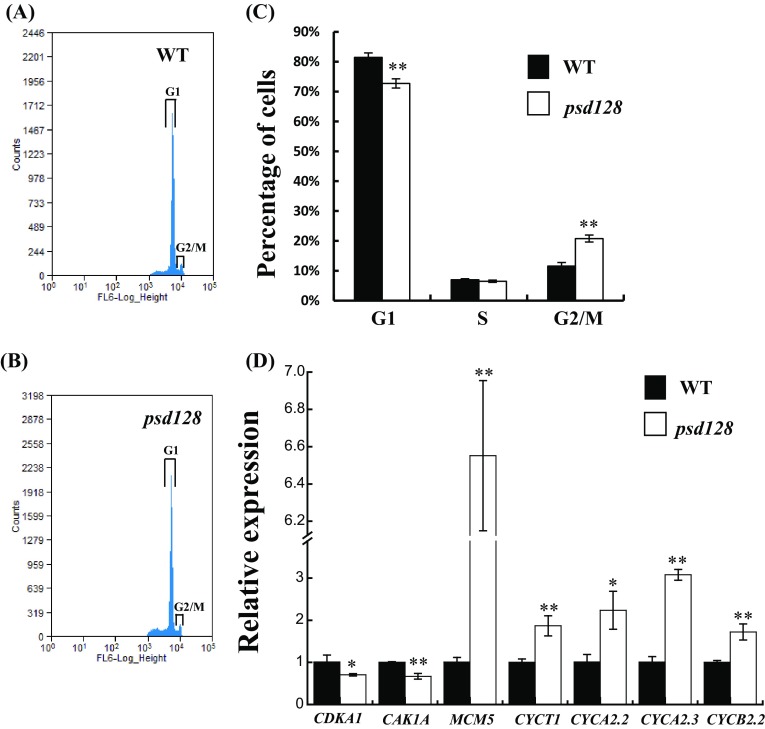
Flow cytometric and cell cycle-related gene expression analysis. **a, b** Flow karyotype histogram of WT (**a**) and *psd128* (**b**), the x-axis represents the relative fluorescence intensity, the y-axis represents the number of cells, and the peaks under the open boxes represent the phases of the cells; **c** Percentage of cells at different phases from 3 day-old shoots in WT and *psd128*. Significance at **P ≤ 0.01 by Student’s *t*-test; **d** relative expression levels of cell cycle related genes in 3 day-old shoots of WT and *psd128*. Values are means ± *SD* from three biological replicates. Asterisks indicate significance by Student’s *t*-test (*P ≤ 0.05, **P ≤ 0.01)

The yeast *CDC48* mutant is defective in the cell cycle (Moir et al. 1982). To determine whether the mutation of *OsCDC48* affected the progression of cell cycle, we investigated the cell status in the cell cycle using shoot suspension cells by flow cytometry. The results revealed that the number of G1 cells in *psd128* was significantly lower than that of WT, and the number of G2/M cells in *psd128* was significantly higher than that of WT while the number of cells in S phase was similar between *psd128* and WT (Fig. 5a–c). In addition, the cell size was similar between *psd128* and WT while the cell number of internode VI in *psd128* was significantly lower than that of WT at the heading stage (Fig. S5A–C). The results demonstrated that *OsCDC48* was involved in regulation of cell cycle progression and the mutation affected both the interphase and cell division. We then determined the expression levels of 7 cell cycle-related genes in WT and *psd128* plants using qRT-PCR. Our results showed that the expression levels of G1-related *CDKA1* and *CAK1A* in *psd128* were significantly down-regulated by 30.28 and 33.06%, respectively, and the expression levels of G1-related *MCM5* and *CYCT1* in *psd128* were up-regulated by 5.55-fold and 86.51%, respectively, compared with WT (Fig. 5d). Furthermore, the expression levels of G2-related *CYCA2.2, CYCA2.3* and *CYCB2.2* in *psd128* were significantly up-regulated by 1.22-fold, 2.07-fold and 71.97%, respectively, compared to WT (Fig. 5d). In addition, we further determined the expression of cell cycle-related genes in overexpressing T_1_ lines. Interestingly, the results exhibited that the expression levels of most these genes were significantly altered but with different patterns compared with *psd128* (Fig. S4C–I). Taken together, we concluded that *OsCDC48* played a critical role in the cell cycle by regulating the expression of a number of cell cycle-associated genes in rice.

### C-terminal deletion affects the expression of diverse genes genome-wide

To investigate the potential roles and mechanisms associated with *OsCDC48*, we carried out high throughput mRNA sequencing (RNA-Seq). The cDNA libraries were prepared from the leaves of 3 WT individual plants and three BC_2_F_3_–psd128 lines (mutant-like) derived from *psd128*/IR64//IR64, respectively. Our results revealed that a total of 374 differentially expressed genes (DEGs) were identified between BC_2_F_3_–psd128 and WT (Supplementary Data S1, total). Among them, 232 genes were up-regulated and 142 genes were down-regulated in BC_2_F_3_–psd128. The cell cycle-associated genes including *CYCA2.3* (*Os01g0233500*), *CYCT1* (*Os02g0438200*), *MCM5* (*Os02g0797400*), *CYCB2.2* (*Os06g0726800*) and *CYCA2.2* (*Os12g0502300*) were significantly up-regulated by 2.40, 1.47, 3.78, 1.32 and 1.52 fold, respectively (Supplementary Data S1, up-regulated). *CDKA1* (*Os03g0118400*) and *CAK1A* (*Os06g0334400*) were significantly down-regulated by 1.11 and 1.02 fold, respectively (Supplementary Data S1, down-regulated). The results were similar to those of qRT-PCR. In addition, a total of 615 genes were expressed only in WT, and 2190 genes covering a set of AAA-ATPase-encoding genes were expressed only in BC_2_F_3_–psd128 (Table S4). Furthermore, the AAA-ATPase encoding genes including *OsAAA-ATPase1* (*Os01g0297200*), *OsAAA-ATPase4 (Os12g0431100), OsAAA-ATPase5* (*Os12g046800*), *OsAAA-ATPase6* (*Os12g0639200*) and *OsAAA-ATPase7* (*Os05g0588850*) were significantly up-regulated by 1.13, 2.90, 2.62, 1.41 and 3.92 fold in BC_2_F_3_–psd128, respectively (Supplementary Data S1, up-regulated). *OsCDC48* (*Os03g0151800*) and *OsCDC48E* (*Os10g0442600*) were significantly down-regulated by 1.38 and 1.51 fold in BC_2_F_3_–psd128, respectively (Supplementary Data S1, down-regulated). The expression levels of *OsAAA-ATPase2* (*Os07g0192800*), *OsAAA-ATPase3* (*Os07g0517800*) and other AAA-ATPase associated genes were similar between WT and BC_2_F_3_–psd128 (Supplementary Table 4). Again, the results were similar to those of qRT-PCR. Taken together, the C terminal mutation affected the cell cycle progression and the global AAA-ATPase activity.

We then performed Gene Ontology (GO) analysis to classify the functions of the 374 DEGs identified. The GO term enrichment indicated that these DEGs could be classified into 74 and 44 GO terms under three biological processes with *P* ≤ 0.05 and ≤ 0.01, respectively (Supplementary Data S2, total). Among the 44 highly significant GO terms, 5 terms belong to Functional process, 16 terms belong to Biosynthetic process, and 23 terms belong to Component process (Supplementary Data S2, total). For the 232 up-regulated genes, a total of 40 GO terms were assigned, and 13 out 40 terms were highly significant terms such as genes that were associated with cell wall biogenesis/organization and cell division (Supplementary Data S2, up-regulated). For the 142 down-regulated DEGs, a total of 89 GO terms were assigned, and 66 out 89 terms were highly significant terms covering genes that were associated with chloroplast biogenesis/development, chlorophyll biosynthesis and photosynthesis (Supplementary Data S2, down-regulated). The results further suggested that *OsCDC48* was involved in the cell cycle and chloroplast development in rice.

To further explore the biological pathways in which the mutation may be involved, we performed KEGG enrichment analysis for the 374 DEGs between WT and BC_2_F_2_–psd128. The results showed that all the DEGs were classified into 8 pathways (Supplementary Data S3, total). The up-regulated 232 DEGs were significantly enriched in 3 pathways including phenylpropanoid biosynthesis, phenylalanine metabolism and glutathione metabolism (Supplementary Data S3, up-regulated), while the 142 down-regulated DEGs were grouped into 6 predominant pathways including photosynthesis-antenna proteins, porphyrin and chlorophyll metabolism, thiamine metabolism, metabolic pathway, photosynthesis, biosynthesis of secondary metabolites (Supplementary Data S3, down-regulated). The results suggested that the C terminal mutation of *OsCDC48* affected multiple pathways including amino acid metabolism probably required for cellular protection and photosynthesis-related metabolism.

## C-terminus is required for CDC48/48, CDC48E/48E and CDC48/48E interaction in vivo

**Fig. 6:**
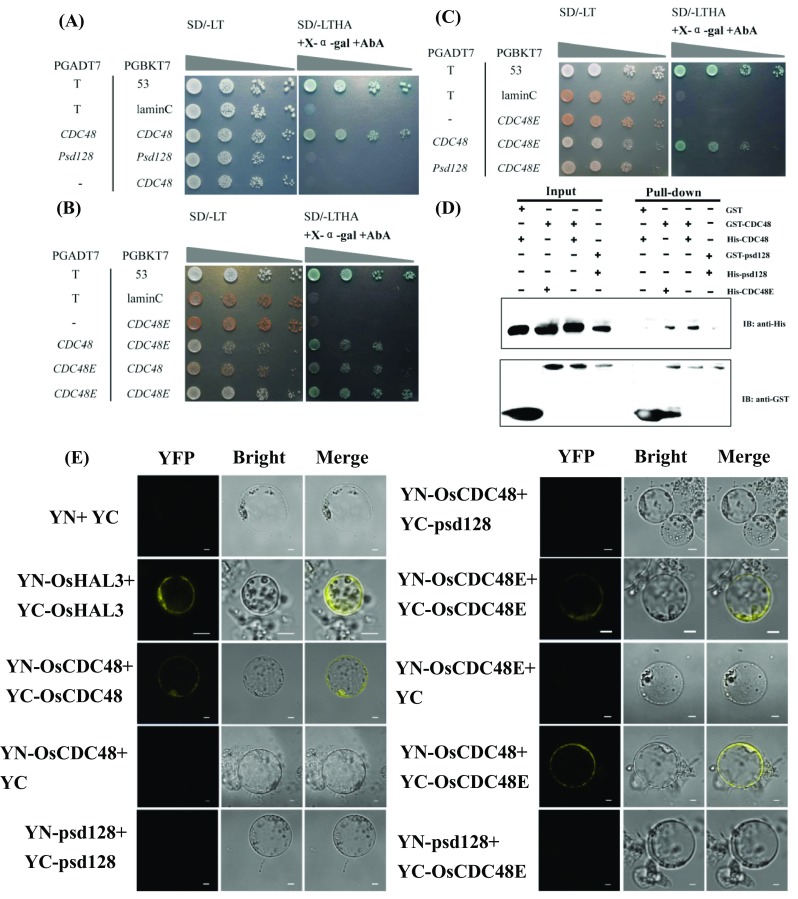
Interaction of OsCDC48 and OsCDC48E in vitro and vivo. **a**–**c** Yeast two-hybrid (Y2H) assay. Serial dilutions (10-fold) of yeast cells expressing the indicated proteins were plated onto SD/-LT nonselective medium (SD/-Leu/-Trp) (Left) and SD/-LTHA selective medium (SD/-Leu/-Trp/-His/-Ade) (Right) supplemented with X-α-gal and AureobasidinA (AbA). pGADT7-T (T) /pGBKT7-53 (53) was used as the positive control and pGADT7-T (T)/pGBKT7-laminC (laminC) was used as the negative control, respectively. **a** Y2H assay shows that OsCDC48 interacts with itself and OsCDC48–PSD128 could not interact with itself; **b** Y2H assay shows that OsCDC48 interacts with OsCDC48E, and OsCDC48E interacts with itself; **c** Y2H assay shows that OsCDC48 interacts with OsCDC48E and OsCDC48–PSD128 could not interact with OsCDC48E; **d** in vitro pull-down assay. The fusion proteins of OsCDC48, OsCDC48–PSD128 and OsCDC48E with a His tag (His-CDC48, His-PSD128 and His-CDC48E) and OsCDC48 and OsCDC48–PSD128 with a GST tag (GST-CDC48, GST-PSD128) were detected by anti-His antibody and anti-GST antibody, respectively; **e** in vivo bimolecular fluorescence complementation assay shows that OsCDC48 and OsCDC48E interacts with itself, respectively, but OsCDC48–PSD128 does not interact with itself, and OsCDC48 interacts with OsCDC48E in rice protoplast. Bars = 5 µm. YN + YC indicates negative control; YN-OsHAL3 + YC-OsHAL3 indicates positive control

CDC48/p97, one of the most abundant proteins in eukaryotic cells, is a molecular chaperone evolutionally conserved in plants, yeasts and animals (Wolf and Stolz 2012). It functions as a hexamer although the monomeric form exists (Bègue et al. 2016). To evaluate the biochemical function of OsCDC48/48E and the effect of C-terminal deletion on OsCDC48 function, we first performed a yeast two-hybrid (Y_2_H) assay with the full-length versions of OsCDC48, OsCDC48E and the truncated OsCDC48–PSD128. Our results revealed that OsCDC48 interacted with OsCDC48 itself, and OsCDC48E interacted with OsCDC48E itself as well. Interestingly, OsCDC48 could interact with OsCDC48E. However, the truncated OsCDC48–PSD128 interacted neither with OsCDC48–PSD128 itself nor with OsCDC48 and OsCDC48E, respectively (Fig. 6a–c). Then, we carried out a pull-down assay to validate the interactions among OsCDC48, OsCDC48E and OsCDC48–PSD128 in vitro. The results revealed that both OsCDC48 and OsCDC48E could interact with itself, and OsCDC48 could interact with OsCDC48E while no interaction was detected between OsCDC48–PSD128 molecules (Fig. 6d). Finally, we performed a bimolecular fluorescence complementation (BiFC) assay in vivo. Consistent with Y2H, the BiFC assay showed that both OsCDC48 and OsCDC48E could interact with itself and each other, while OsCDC48–PSD128 could not interact with OsCDC48, OsCDC48E and itself (Figs. 6e, S6). Taken together, these results indicated that OsCDC48/48E was likely functioning as a hetero-oligomers and the C-terminus played a crucial role for the interaction.

### OsCDC48E knockout plants exhibit premature senescence and death phenotype

**Fig. 7:**
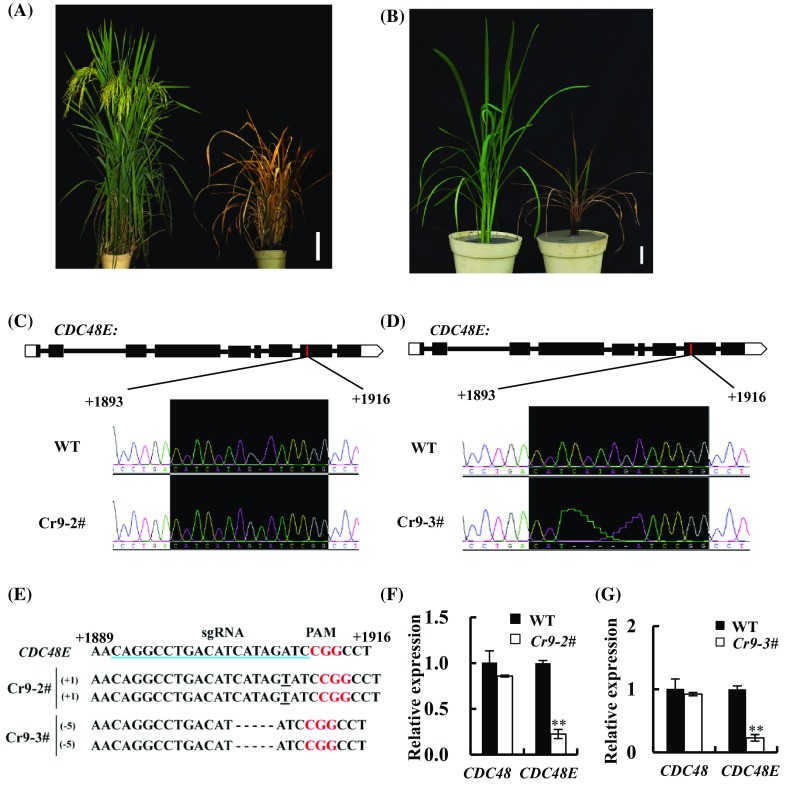
Phenotypic characterization and expression analysis of OsCDC48E-knock out lines. **a** Phenotype of WT (left) and knock out plant Cr9-2# (right) at the heading stage. Bar = 5 cm; **b** phenotype of WT (Left) and knock out plant Cr9-3# (right) at the tillering stage. Bar = 5 cm; **c**, **d** structure of *OsCDC48E*: the empty box indicates 5′UTR, black boxes indicate exons, lines indicate introns and white arrows indicate 3′UTR and the red transverse lines indicate nucleotide mutations in Cr9-2# and Cr9-3#, respectively. The last 3 nucleotides in the black shadow area are PAM sequence (CGG); **e** mutations in Cr9-2# and Cr9-3#. Cr9-2# is a homozygous mutant carrying a T insertion and Cr9-3# is a homozygous mutant carrying a 5-bp deletion. The sgRNA target sequence is underlined in blue and the PAM motif is highlighted in red letters; **f***OsCDC48* and *OsCDC48E* expression levels in WT and Cr9-2# at the heading stage; **g***OsCDC48* and *OsCDC48E* expression levels in WT and Cr9-3# at the tillering stage. Values are means ± *SD* from three biological replicates. Significance at **P ≤ 0.01 by Student’s *t*-test

Having established that OsCDC48E was required for the interaction with OsCDC48, we speculated that OsCDC48E alone must be essential for plant survival in rice. To evaluate this possibility, we knocked out *OsCDC48E* in the wild-type (IR64) background using the CRISPR/Cas9 approach (Wang et al. 2015). The results showed that a total of 9 positive transformants (T_0_) were obtained and all of them exhibited dwarfism, premature senescence and death before heading, mimicking the *psd128* phenotype although *psd128* was able to set a few seeds before completely died (Fig. 7; Table S3). Among the 9 CRISPR-Cas9 knockout plants, *Cr9-2#* and *Cr9-3#* displayed both premature senescence phenotype and shorter plant height at the heading and tillering stages, respectively (Fig. 7a, b; Fig S7A). *Cr9-2#* was a homozygous mutant carrying a T nucleotide insertion in the 8th exon (Fig. 7c, e) while *Cr9-3#* was a homozygous mutant carrying a 5-bp (CATAG) deletion in the targeted 8th exon (Fig. 7d, e). As expected, the expression levels of *OsCDC48E* in Cr9-2# and Cr9-3# were significantly reduced compared to WT (Fig. 7f, g). In addition, Cr9-2# exhibited a premature senescence phenotype with significantly reduced levels of photosynthetic pigments (Fig. S7B) similar to *psd128* (Huang et al. 2016). Furthermore, the remaining 7 *OsCDC48E* knock out plants died at a very young seedling stage (data not shown).We hypothesize that OsCDC48 and OsCDC48E are likely to form a heteromultimeric complex to control plant survival by regulating senescence-associated genes and the cell cycle progression in rice.

## Discussion

### OsCDC48 involves in cell cycle regulation and is essential for rice growth and survival

The cell cycle plays a crucial role in regulating the growth and development of organisms. Defective in the cell cycle would lead to abnormal cell proliferation and differentiation, division and cell death (Hartwell and Weinert 1989). Previous studies have inspected the biological significance of rice genes including *GSN1, DEL1*and *TAD1*, and confirmed that they are involved in the cell cycle progression and ultimately affect the leaf development and senescence, plant height and grain yield (Guo et al. 2018; Leng et al. 2017; Xu et al. 2012). The initiation and establishment of plant branches/tillers is a complex biological process involving a combination of multiple factors (Wang and Li 2011) and is ultimately achieved through the basic biological process of cell division (Shimizu and Mori 1998). In the present study, OsCDC48 appeared to play an important role in the control of leaf senescence, plant development and survival in rice. Overexpression of *OsCDC48* delayed leaf senescence and significantly increased the tiller numbers leading to increased grain yield, suggesting that *OsCDC48* is a potential genetic factor for rice yield improvement. In contrast, overexpression of *OsCDC48–psd128* dramatically reduced the tiller numbers, plant height and caused premature leaf senescence and plant death because of elevated accumulation of H_2_O_2_, indicating that H_2_O_2_ signaling might participate in the control of tillering and plant height. Meanwhile, the level of MDA, an indicator of cell membrane damage, was highly elevated in the *OsCDC48–psd128* overexpression plants which exhibited premature senescence similar to *psd128* (Fig. 4d–m). Our results supported the conclusion that MDA and H_2_O_2_ over production could promote senescence in plant cells (Brodersen et al. 2002). The leaf senescence and survival of *psd128* is likely resulted from abnormal cell cycle progression manifested in G1 and G2/M phases (Fig. 5a–c). Consistent with the flow cytometric results, the expression levels of genes regulating the cell cycle were significant altered in *psd128* (Fig. 5d). In addition, histochemical examination showed that the dwarf phenotype of *psd128* was caused by the shortened internodes resulting from a marked reduction of cell number in *psd128* (Fig. S4), suggesting that cell division was inhibited. Moreover, although the expression of cell cycle-related genes in overexpressing T_1_ lines was significantly altered while the patterns were somewhat different from those of *psd128*, further experiments are necessary to be carried out for clarification (Fig. S5). Nevertheless, our results strongly suggest that *OsCDC48* controls plant growth, development and survival by regulating the cell cycle progression in rice.

### C-terminus of OsCDC48 influences ATPase activity

A typical eukaryotic AAA-ATPase molecular structure contains an N-terminal domain, an AAA domain and a C-terminal domain. The biochemical functions for a broad utility of p97/CDC48 lie in its ATPase activity which is believed to be carried out mainly through the AAA domain by hydrolyzing ATP to provide energy for diverse cellular functions (Peters et al. 1990). The ATPase activity of p97/CDC48 was first demonstrated in *Xenopus laevis* (Peters et al. 1990). The N-terminal domain and C-terminal of p97/CDC48, are also required for modulating ATPase activity via either posttranslational modification or other pathways (Jentsch and Rumpf 2007; Wang et al. 2004). It has been shown that the N-domain position relative to the D1 ring is linked to ATP hydrolysis ability and removal of the C-terminal region reduces ATPase activity probably by affecting the hexamer stability (Niwa et al. 2012). In the present study, *psd128* is defective in the C terminus, thus we focus on whether the ATPase activity is affected with removal of the C-terminal 27 aa residues of OsCDC48 in rice. Interestingly, the total ATPase activities in leaves were significantly increased in *psd128* while the purified OsCDC48–PSD128 exhibited a significantly reduced ATPase activity compared to the WT OsCDC48 protein. Increased total ATPase activity was contributed by other members of ATPase-related genes in *psd128* and *OsCDC48–psd128* overexpression lines. In contrast, increased total ATPase activity in *OsCDC48* overexpression lines was resulted from elevated enzymatic activities of OsCDC48 and OsCDC48E (Fig. 4f). This observation supports that the C terminal defection reduces ATPase activity that is likely associated with hexamer stability which requires to be validated in OsCDC48. Additionally, removal of the C terminal region of *OsCDC48* induced significantly elevated levels of expression of a set of other members of AAA-ATPase genes in *psd128*, however, it could not fully compensate for the role by OsCDC48 as the mutant eventually dies at the heading stage (Huang et al. 2016). The rice genome (cv. Nippobare) harbors 29 members of AAA proteins including CDC48. It would be necessary to investigate the effect of the C terminal defect on all the members so as to provide an insight into the link among the members although a number of AAA-ATPases showing similar expression levels between two genotypes were detected by RNA-seq (Table S5). Besides, the C terminal defect influenced the expression of a large number of genes as shown by RNA-seq between the two genotypes, indicating that the stability of the gene network plays a crucial role at the molecular and cellular levels for rice growth, development and survival. Taken together, we conclude that the C-terminal region of OsCDC48 is critical to plant growth, development and survival via controlling the ATPase activity.

### OsCDC48/48E hetero-oligomeric complex is essential for rice survival

In eukaryotic cells, p97/CDC48 is a highly abundant hexameric AAA-ATPase that functions as a molecular chaperone in diverse cellular activities. The assembly and disassembly of the hexameric p97/CDC48 complex itself is a dynamic process (Paro et al. 2007). The AAA domain is highly conserved evolutionally while both the N domain and C domain are structurally flexible and able to modulate protein–protein interaction (Jentsch and Rumpf 2007; Wang et al. 2004). The main function of the N-domain is to control the binding of cofactors and ubiquitylated protein substrates (Jentsch and Rumpf 2007; Wang et al. 2004), and the conformation of the N-domain in relation to the D1-D2 hexamer is directly linked to ATP hydrolysis (Niwa et al. 2012). The C domain contains a tyrosine phosphorylation site that is thought critical to multiple p97/CDC48 functions (Wang et al. 2003a, b). Moreover, the C domain is also considered necessary for hexamer stability and full ATPase activity (Niwa et al. 2012). In the present study, the most striking finding is that interaction between OsCDC48 (Os03g0151800) and OsCDC48E (Os10g0442600) was required for its function in the control of leaf senescence, growth, development and survival, indicating that OsCDC48/48E may form and function as a heterohexamer (Arlt et al.1996; Rubin et al. 1998; Turner et al.1999). In contrast to previous report, p97/CDC48 has been thought functioning in the homo-hexameric form (Peters et al. 1990; Zhang et al. 2000). Whether OsCDC48/48E form and act as a heterohexamer is still required to be validate structurally, however, several observations in the present study support this speculation. Firstly, the expression pattern of *OsCDC48* and *OsCDC48E* was highly similar, indicating that they might act together and have similar functions or are involved in the same pathways. Secondly, both OsCDC48, and OsCDC48E are localized to the nucleus and cytoplasm, indicating that they might function in the same cellular sites with similar roles in relevant biological processes. Lastly, *OsCDC48E* knockout plants exhibit similar phenotype to *psd128* especially in terms of premature senescence and plant lethality, indicating an irreplaceable role by OsCDC48E and only presence of both of them could secure the normal growth, development and senescence. Taken together, the results suggest that OsCDC48 and its homologue OsCDC48E might coordinate in function to regulate plant growth and development.

## Electronic supplementary material

Below is the link to the electronic supplementary material.

Supplementary material 1 (DOCX 10697 KB)
