# Supplementary material for: OsCDC48/48E complex is required for plant survival in rice (Oryza sativa L.)
Source: Plant Mol Biol. 2019 Apr 1;100(1):163–79. doi: 10.1007/s11103-019-00851-9 (PMC6513905; doi:10.1007/s11103-019-00851-9)
Supplement: Supplementary file 1 — Supplementary material 1 (DOCX 10697 KB) [file 11103_2019_851_MOESM1_ESM.docx]

**Supplementary data:**


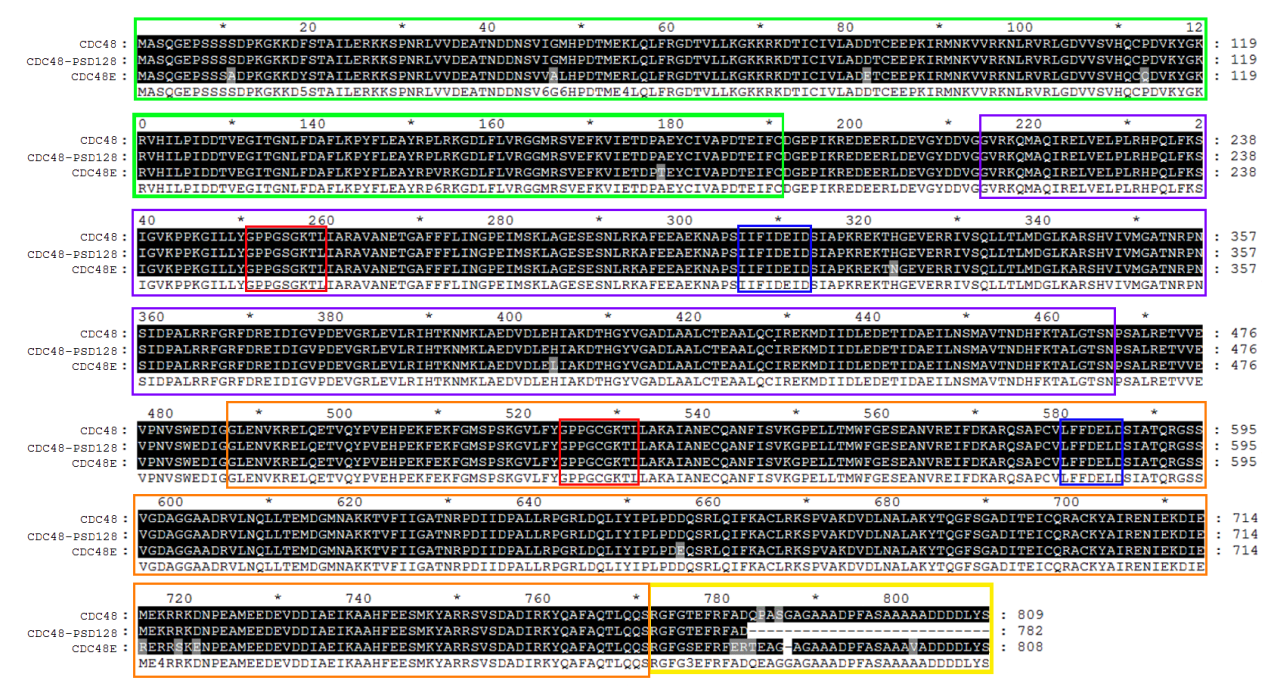


**(B)**


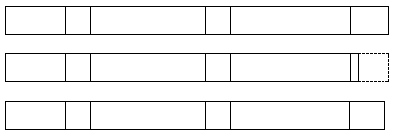


**CDC48**

**N**

**L1**

**ATPase D1**

**L2**

**ATPase D2**

**C**

**CDC48-PSD128**

**N**

**L1**

**ATPase D1**

**L2**

**ATPase D2**

**C**

**CDC48E**

**N**

**L1**

**ATPase D1**

**L2**

**ATPase D2**

**C**

**1**

**191**

**213**

**466**

**486**

**771**

**809**

**1**

**191**

**213**

**466**

**486**

**771**

**808**

**1**

**191**

**213**

**466**

**486**

**771**

**782**

**(A)**

**Figure S1.** Structural comparison of OsCDC48, OsCDC48-PSD128 and OsCDC48E. **a** Structures of CDC48, CDC48-PSD128, and CDC48E. The N-terminus (N) consists of 1-199 aa; two ATPase domains are D1 (213-466 aa) and D2 (486-770 aa); Link 1 (L1) is located between the N-terminus and D1; Link 2 (L2) is located between D1 and D2; The C-terminus (C) varies: C of OsCDC48 is 771-809 aa; C of CDC48-PSD128 has a deletion (771-782 aa, dotted line stand for the deletion); C of OsCDC48E has one aa less than that of OsCDC48 (771-808 aa). **b** The sequence alignment among CDC48, CDC48-PSD128 and CDC48E. The N-terminus, green box; D1, purple box; D2, orange box; Walker A, red box; Walker B, blue box. The C-terminus, yellow box. OsCDC48 shares 97.03% amino acid identity with OsCDC48E.


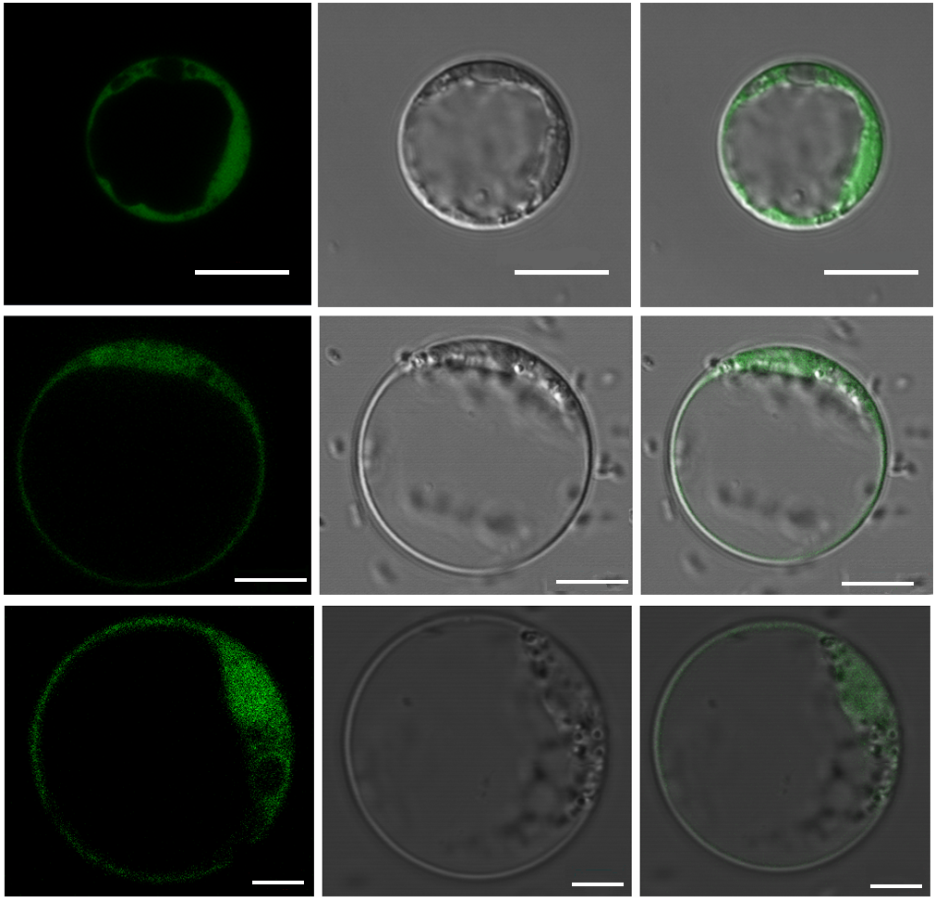


**35S::GFP**

**35S::OSCDC48-GFP**

**35S::OsCDC48-PSD128-GFP**

**GFP**

**Bright field**

**Merged**

**(A)**

**(B)**

**(C)**

**(D)**

**(E)**

**(F)**

**(G)**

**(H)**

**(I)**

**Figure S2.** Subcellular localization of OsCDC48-GFP and OsCDC48-PSD128-GFP.  **a-c** GFP signal in tissues transformed with the empty GFP vector. **d-f** GFP signal in tissues transformed with the OsCDC48-GFP fusion protein. **g-i** GFP signal in tissues transformed with OsCDC48-PSD128-GFP fusion protein. (**a) (d) (g)**: GFP signals; (**b) (e) (h)**: bright-field images and (**c) (f) (i)**: the merged images. Bars = 10 μm.


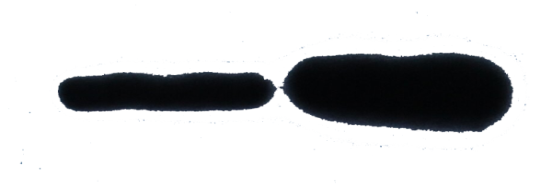


**OsCDC48**

**OsCDC48-PSD128**

**(B)**


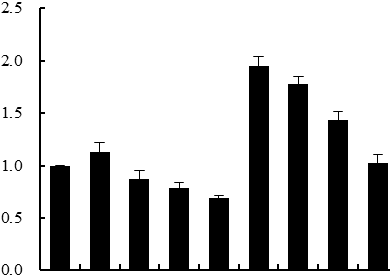

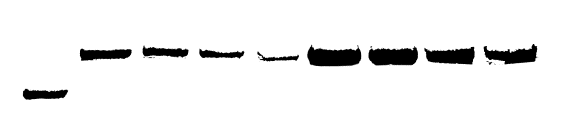


OsCDC48

OsCDC48-PSD128

Relative Concentration（μg/μl）

**(C)**

**BSA**

**24**

**20**

**15**

**10**

**24**

**20**

**15**

**10**

**55**

**100**

**70**

**170**

**kDa**


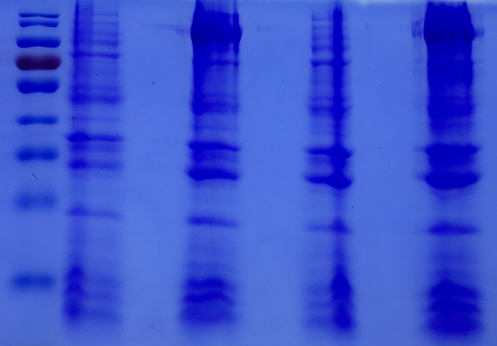


**M**

**-IPTG**

**+IPTG**

**-IPTG**

**+IPTG**

**OsCDC48**

**OsCDC48-PSD128**

**(A)**

**15**

**25**

**35**

**40**

**130**

**1**

**2**

**3**

**4**

**5**

**6**

**7**

**Figure S3.** Fusion protein expression and concentration of the purified OsCDC48 and OsCDC48-PSD128. **a** Induction expression for 2 hours by 1mmol/L IPTG.**M**, Protein Marker; Lane 1 and 3 are OsCDC48; 5 and 7 are OsCDC48-PSD128; 2, 4, and 6 are empty lanes. **-IPTG**, expression without IPTG induction; **+IPTG**, expression of pET28a with IPTG induction; Red arrows stand for the target protein bands; **b** Western blot of purified proteins. Purified OsCDC48 and OsCDC48-PSD128 were analyzed by western blot with anti-6×His antibody; **c** SDS-PAGE of purified protein. From left to right lanes, control fraction (Control BSA, 1 µg/µl) and the purified fractions (CDC48: 24µl, 20µl, 15µl, 10µl; PSD128: 24µl, 20µl, 15µl, 10µl) were run in 10% SDS-PAGE and visualized by Coomassie brilliant blue staining (top). The histogram shows the relative concentration of the purified fractions by Image J (bottom).

*

*

*

*

**Relative expression**

**WT**

**L3**

**L5**

**L2**

**L4**


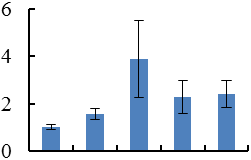


***CAK1A***

**(C)**

*

*

**Relative expression**

**WT**

**L3**

**L5**

**L2**

**L4**


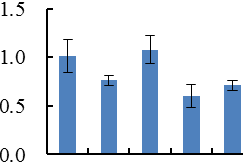


***CDKA1***

**(D)**

**

**

**

**

**Relative expression**

**WT**

**L3**

**L5**

**L2**

**L4**


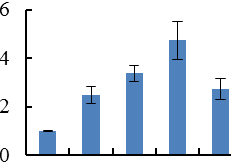


***MCM5***

**(E)**

**

**

**

**

**Relative expression**

**WT**

**L3**

**L5**

**L2**

**L4**


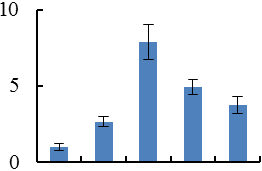


***CYCA2.2***

**(F)**

*

**

**Relative expression**

**WT**

**L3**

**L5**

**L2**

**L4**


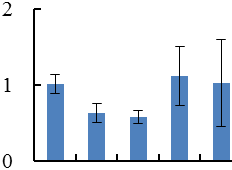


***CYCA2.3***

**(G)**

*

*

**Relative expression**

**WT**

**L3**

**L5**

**L2**

**L4**


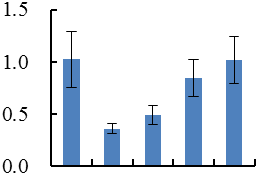


***CYCB2.2***

**(H)**

**

**

*

**

**Relative expression**

**WT**

**L3**

**L5**

**L2**

**L4**


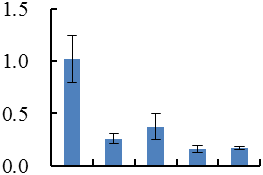


***CYCT1***

**(I)**

**(A)**

***Osh36***

*

*

**

**

**Relative expression**

**WT**

**L3**

**L5**

**L2**

**L4**


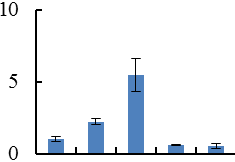


***OsI57***

**

**

**

**

**Relative expression**

**WT**

**L3**

**L5**

**L2**

**L4**

**(B)**


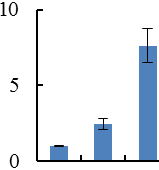


**Figure S4** Relative expression levels of senescence associated genes **(a**-**b)** and cell cycle related genes **(c-i)** in 15-day-old seedlings of overexpression T_1_ lines. Values are means ± SD from three biological replicates. Asterisks indicate significance by Student’s *t*-test (* P ≤ 0.05, **P ≤ 0.01).


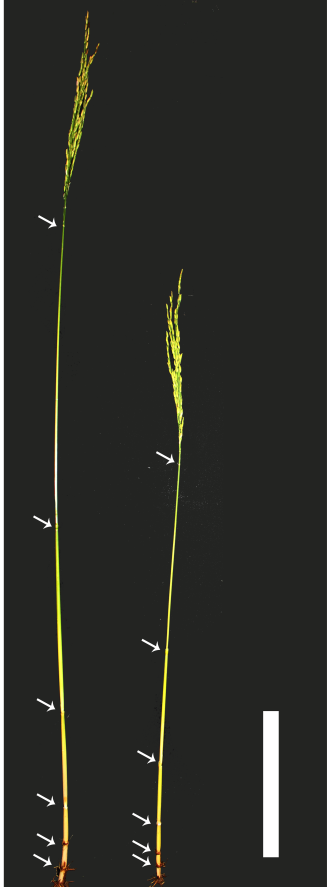

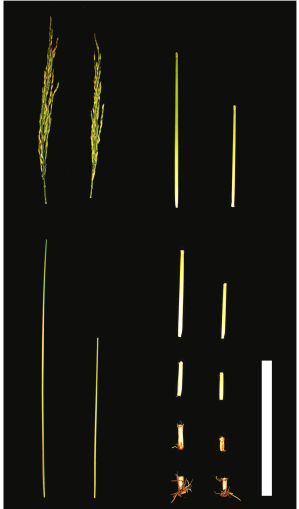


**P**

**Ⅰ**

**Ⅱ**

**Ⅲ**

**Ⅳ**

**Ⅴ**

**Ⅵ**


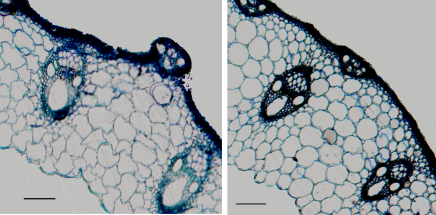


**WT**

***psd128***

**Internode length (cm)**


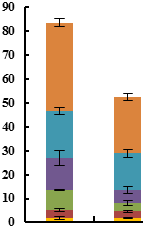


**

**

**Ⅰ**

**Ⅱ**

**Ⅲ**

**Ⅳ**

**Ⅴ**

**Ⅵ**


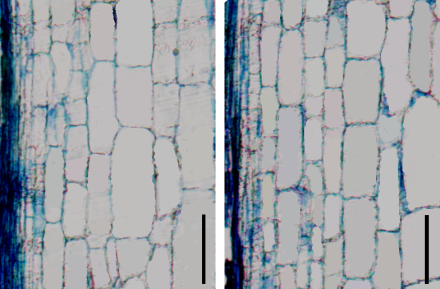

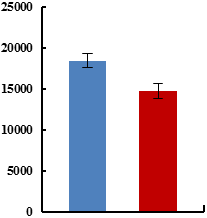


**NO. of cell in y-axis**

***psd128***

**WT**

**

**(A)**

**(B)**

**(C)**

**(D)**

**(E)**

**(F)**

**(G)**

**(H)**

*

*

**Figure S5** Comparison of internode lengths between WT and *psd128* at the heading stage. **a** Leading culms of WT (left) and *psd128* (right). Arrows indicate the position of nodes. Bar = 20 cm. **b** and **c** Panicles and internodes on the leading culm of WT (left) and *psd128* (right).Ⅰ, first internode from bottom; Ⅱ, second internode; Ⅲ, third internode; Ⅵ, fourth internode; Ⅴ, fifth internode; Ⅵ, sixth internode; P, panicle. Bar = 20 cm; **d** and **e** Cross-section of internode Ⅵ of WT (left) and *psd128* (right) in mature plants, Bar = 20 μm. Longitudinal sections: **f** WT and **g** *psd128* internode Ⅵ, Bar = 20 μm; **h** Cell numbers in internode Ⅵ. Data in **c** and **h** are means ± SD from three biological replicates. Asterisks indicate significance between WT and *psd128* by Student’s *t*-test (* *P* ≤ 0.05; ** *P* ≤ 0.01).

YN+YC

YN-CDC48+

YC-CDC48

YN-CDC48E+

YC-CDC48E

YN-CDC48+

YC-CDC48E

YFP

Bright field

Merged


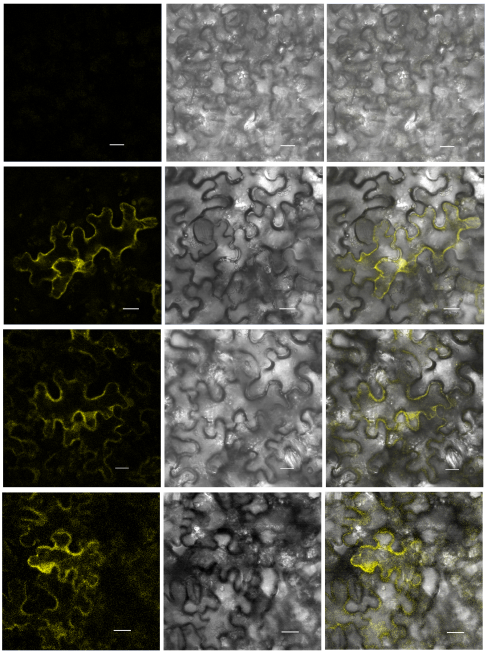


YN-OsHAL3+

YC- OsHAL3

YN-Psd128+

YC- Psd128

YN-CDC48E+

YC- Psd128

YN-CDC48+

YC-Psd128

YFP

Bright field

Merged


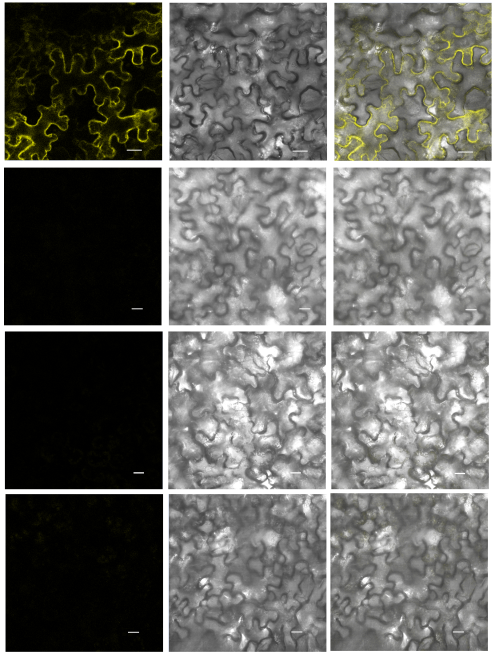


**Figure S6** BIFC assay for interaction of OsCDC48 and OsCDC48E in tobacco mesophyll cells. YN+YC indicates the negative control; YN-OsHAL3+YC-OsHAL3 indicates the positive control. Bar = 20 μm.


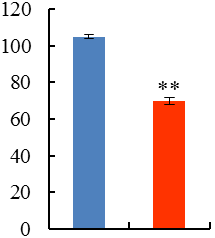


**WT**

***Cr9-2#***

**Plant height (cm)**

**(A)**


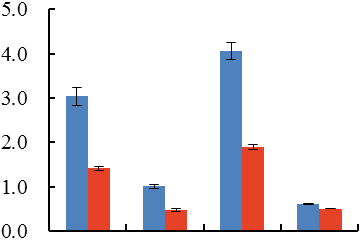


**Content (mg/g)**

**Chla**

**Chlb**

**Chl**

**Car**

**

**

**

**

**WT**

***Cr9-2#***

**(B)**

**Figure S7** Comparison of plant height, Chlorophyll and carotenoid contents between WT and CRISPR/Cas9 knock out line *Cr9-2#* at the heading stage.

**a** Plant height of WT (left) and *Cr9-2#* (right); **b** Chlorophyll a (Chl a), Chl b, total Chl, and Carotenoid (Car) content of WT and *Cr9-2#* . Data in (**a**) and (**b**) are means ± SD from three biological replicates. ** indicates significance at P ≤ 0.01 by Student’s *t*-test.

**Table S1.** Primers used in this study.

| Experiment | Primer name | Sequence (5’ to 3’) |
| --- | --- | --- |
| **Subcellular localization** | pAN580-CDC48-F | GCCCAGATCAACTAGTATGGCGAGCCAGGGAGAGCC |
|  | pAN580-CDC48-R | TGCTCACCATGGATCCACTATATAAATCATCATCGT |
|  | pAN580-Psd128-F | GCCCAGATCAACTAGTATGGCGAGCCAGGGAGAGCC |
|  | pAN580-Psd128-R | TGCTCACCATGGATCCGTCAGCGAACCTAAACTCGG |
|  | pAN580-CDC48E-F | GCCCAGATCAACTAGTATGGCGAGCCAGGGGGAGCC |
|  | pAN580-CDC48E-R | TGCTCACCATGGATCCACTATATAAATCGTCATCAT |
|  | 163-Mcherry-D53-F | AGGACAGCCCAAGCTTATGCCCACTCCGGTGGCCGC |
|  | 163-Mcherry-D53-R | TGCTCACCATGGATCCACAATCTAGAATTATTCTTG |
|  | 163-Mcherry-TAD1-F | AGGACAGCCCAAGCTTATGGATCACCACCACCACCA |
|  | 163-Mcherry-TAD1-R | TGCTCACCATGGATCCCCGGATGTAGCTCCTAACAA |
| **Overexpression vector construction** | 1305-GFP-CDC48-F | CGGAGCTAGCTCTAGAATGGCGAGCCAGGGAGAGCC |
|  | 1305-GFP-CDC48-R | TGCTCACCATGGATCCACTATATAAATCATCATCGT |
|  | 1305-GFP-Psd128-F | CGGAGCTAGCTCTAGAATGGCGAGCCAGGGAGAGCC |
|  | 1305-GFP-Psd128 -R | TGCTCACCATGGATCCGTCAGCGAACCTAAACTCGG |
| **Protein expression** | pET28a-CDC48-F | CGCGCGGCAGCCATATGATGGCGAGCCAGGGAGAGCC |
|  | pET28a-CDC48-R | TGCTCGAGTGCGGCCGCCTAACTATATAAATCATCA |
|  | pET28a-Psd128-F | CGCGCGGCAGCCATATGATGGCGAGCCAGGGAGAGCC |
|  | pET28a-Psd128-R | TGCTCGAGTGCGGCCGCCTAGTCAGCGAACCTAAACA |
|  | pET28a-CDC48E-F | CGCGCGGCAGCCATATGATGGCGAGCCAGGGGGAGCC |
|  | pET28a-CDC48E-R | TGCTCGAGTGCGGCCGCCCTAACTATATAAATCGTCAT |
|  | pGEX4T-1-CDC48-F | GGTTCCGCGTGGATCCATGGCGAGCCAGGGAGAGCC |
|  | pGEX4T-1-CDC48-R | GTCGACCCGGGAATTCCTAACTATATAAATCATCA |
|  | pGEX4T-1-Psd128-F | GGTTCCGCGTGGATCCATGGCGAGCCAGGGAGAGCC |
|  | pGEX4T-1-Psd128-R | GTCGACCCGGGAATTCCTAGTCAGCGAACCTAAACA |
| **Yeast two-hybrid** | pGADT7-CDC48-F | GCCATGGAGGCCAGTGAATTCATGGCGAGCCAGGGAGAGC |
|  | pGADT7-CDC48-R | AGCTCGAGCTCGATGGATCCCCTAACTATATAAATCATCA |
|  | pGADT7-Psd128-F | GCCATGGAGGCCAGTGAATTCATGGCGAGCCAGGGAGAGC |
|  | pGADT7-Psd128-R | AGCTCGAGCTCGATGGATCCCCTAGTCAGCGAACCTAAACA |
|  | pGADT7-CDC48E-F | GCCATGGAGGCCAGTGAATTCATGGCGAGCCAGGGGGAGC |
|  | pGADT7-CDC48E-R | AGCTCGAGCTCGATGGATCCCCTAACTATATAAATCGTCAT |
|  | pGBKT7-CDC48-F | TGGCCATGGAGGCCGAATTCATGGCGAGCCAGGGAGAGC |
|  | pGBKT7-CDC48-R | ATGCGGCCGCTGCAGGTCGACGCTAACTATATAAATCATCA |
|  | pGBKT7-Psd128-F | TGGCCATGGAGGCCGAATTCATGGCGAGCCAGGGAGAGC |
|  | pGBKT7-Psd128-R | ATGCGGCCGCTGCAGGTCGACGCTAGTCAGCGAACCTAAAC |
|  | pGBKT7-CDC48E-F | TGGCCATGGAGGCCGAATTCATGGCGAGCCAGGGAGAGC |
|  | pGBKT7-CDC48E-R | ATGCGGCCGCTGCAGGTCGACGCTAACTATATAAATCGTCAT |
| **BIFC assay** | 1300S-YN-CDC48-F | CTGAGGAGGATCTTCCCGGGATGGCGAGCCAGGGAGAGCC |
|  | 1300S-YN-CDC48-R | GGGCATGCCTGCAGGTCGACCTAACTATATAAATCATCAT |
|  | 2300S-YC-CDC48-F | CTAGGAGCTCGGTACCCGGGATGGCGAGCCAGGGAGAGCC |
|  | 2300S-YC-CDC48-F | TCGTATGGGTACATACTAGTACTATATAAATCATCATCGT |
|  | 1300S-YN-Psd128-F | CTGAGGAGGATCTTCCCGGGATGGCGAGCCAGGGAGAGCC |
|  | 1300S-YN- Psd128-R | GGGCATGCCTGCAGGTCGACCTAGTCAGCGAACCTAAACT |
|  | 2300S-YC- Psd128-F | AGGAGCTCGGTACCCGGGATGGCGAGCCAGGGAGAGCC |
|  | 2300S-YC- Psd128-F | ATGGGTACATACTAGTGTCAGCGAACCTAAACTCGG |
|  | 1300S-YN-CDC48E-F | GGAGGATCTTCCCGGGATGGCGAGCCAGGGGGAGCC |
|  | 1300S-YN-CDC48E -R | ATGCCTGCAGGTCGACCTAACTATATAAATCGTCAT |
|  | 2300S-YC- CDC48E -F | AGGAGCTCGGTACCCGGGATGGCGAGCCAGGGGGAGCC |
|  | 2300S-YC- CDC48E -R | ATGGGTACATACTAGTACTATATAAATCGTCATCAT |
| **CRISPR/ Cas9** | CRISPR-CDC48E-F | GGCA CAGGCCTGACATCATAGATC |
|  | CRISPR-CDC48E-R | AAAC GATCTATGATGTCAGGCCTG |
|  | Test-Cr9-F | CTGAATCAGCTGTTGACAGA |
|  | Test-Cr9-R | GATTAGTTTCAACACAAAGGCAT |
| **qRT-PCR** | qRT-PCR-Ubi-F | GCTCCGTGGCGGTATCAT |
|  | qRT-PCR-Ubi-R | CGGCAGTTGACAGCCCTAG |
|  | qRT-PCR-CDC48-F | GAATGCTCTTGCCAAATACACC |
|  | qRT-PCR -CDC48-R | TCCTCCGCTTCTCCATCTCG |
|  | qRT-PCR -CDC48E-F | AAGCAGAGAAGAATGCACCA |
|  | qRT-PCR -CDC48E-R | TGAAACAATACGACGTTCCACT |
|  | CDKA1-F | GGTTTGGACCTTCTCTCTAAAATGC |
|  | CDKA1-R | AGAGCCTGTCTAGCTGTGATCCTT |
|  | CAK1A-F | GACCGACAAGGGTTTCAGCAT |
|  | CAK1A-R | CCAGCATGTTCAGGAAGATACAAT |
|  | MCM5-F | AAGGAGAACTGCCTGTCCATGA |
|  | MCM5-R | AGTGGCCTTAGCTTTCACCCTC |
|  | CYCT1-F | GCATTTGTTGCAGCTCAAG |
|  | CYCT1-R | TCACCACTTCGCTGACTTATTG |
|  | CYCA2.2-F | AGGTTGTCAAGATGGAGAGCGA |
|  | CYCA2.2-R | CGCTTTTTGTCTTCCTGGCA |
|  | CYCA2.3-F | GTTTCGGTTGACGAGACGATGT |
|  | CYCA2.3-R | CGCTGCAAGGAACCTAGAACTG |
|  | CYCB2.2-F | CTCAAGGCTGCACAATCTGACA |
|  | CYCB2.2-R | GCATTGACGGCTGGAATTTG |
|  | Osh36-F | GCACGGAGGCGAACGA |
|  | Osh36-R | TTGAGCGGTAGCACCCATT |
|  | OsI57-F | ACCCTAAAGTAAATGAAGTC |
|  | OsI57-R | CCTGCTCTTGTCTTGTTA |
|  | Os01g0297200-RT-F | GGCGACTCCTTCCGGTACGAT |
|  | Os01g0297200-RT-R | CTCCCTCTTCCTGGCCGTGTCC |
|  | Os07g01928000-RT-F | ACTCCTACCTCCCGCACGTCT |
|  | Os07g01928000-RT-R | CCACGTAGCTCCACGCCGACT |
|  | Os07g0517800-RT-F | CCGGCCACGTTCGACACCCTC |
|  | Os07g0517800-RT-R | CGCTTCCACGCCTTGCCGAT |
|  | Os12g0467700-RT-F | GCTGCTGTTCACCAACCACT |
|  | Os12g0467700-RT-R | TCTCAACCTTCTTGGCATGGTC |
|  | Os12g0468000-RT-F | GCTGCTGTTCACCAACCACT |
|  | Os12g0468000-RT-R | TCTCAACCTTCTTGGCATGGTC |
|  | Os12g0639200-RT-F | AAGCATCCAAGGTCACGCTCT |
|  | Os12g0639200-RT-R | GGTCCAGCTTCTCCTTGTGGT |
|  | Os05g0588850-RT-F | ATGCCATGTACCCAGAAATCGAG |
|  | Os05g0588850-RT-R | GGGTTCATTCCTCTTTGCCTT |

**Table S2** Performance of agronomic traits of WT and OsCDC48 overexpression lines

| Material | Tiller Number/plant | Seed setting rate (%) | Grain Number/Tiller | 1000 Grain weight (g) | Plant height (cm) | Grain yield/Plant (g) |
| --- | --- | --- | --- | --- | --- | --- |
| IR64 | 22.1±3.9 ^a^ | 54.9±1.7^a^ | 120.4±6.9 ^a^ | 26.4±0.4^a^ | 110.3±1.9^a^ | 38.8±8.9 ^a^ |
| OX-CDC48-L2 | 36.0±2.1 ^b^ | 58.4±3.1^a^ | 121.9±4.1 ^a^ | 25.0±0.3^b^ | 109.6±2.1^a^ | 64.2±7.4 ^b^ |
| OX-CDC48-L4 | 35.1±1.2 ^b^ | 56.0±3.3^a^ | 126.8±4.7 ^a^ | 25.2±0.3^b^ | 110.2±2.6^a^ | 63.0±6.3 ^b^ |

Different letters after each number indicate significant difference at *p* ≤ 0.01 by Duncan’s. OX, overexpression

**Table S3** Performance of agronomic traits of *psd128* and CRISPR/Cas9 knock out plants

| Material | Tiller Number/plant | Seed setting rate (%) | Grain Number/Tiller | 1000 Grain weight (g) | Plant height (cm) | Grain yield/Plant (g) |
| --- | --- | --- | --- | --- | --- | --- |
| IR64 | 22.3±0.6 ^a^ | 53.7±1.5^a^ | 122.7±5.0 ^a^ | 26.1±0.3^a^ | 103.0±2.0^a^ | 38.3±1.6 ^a^ |
| *psd128* | 13.3±1.5 ^b^ | 18.7±3.1^b^ | 61.7±3.5 ^b^ | 16.2±1.2^b^ | 71.6±0.8^b^ | 2.5±0.7 ^b^ |
| *Cr9-2#* | 10.3±1.5 ^b^ | NV | NV | NV | 70.7±3.3^b^ | NV |
| *Cr9-3#* | NV | NV | NV | NV | NV | NV |

NV stands for not available because of plant lethality. Different letters after each number indicate significance at *P* ≤ 0.01 by Duncan’s

**Table S4.** AAA-ATPase related genes were expressed only in *psd128*

| Gene Identifier | Description |
| --- | --- |
| OS01G0641800 | AAA-family ATPase, putative, expressed |
| OS02G0537400 | AAA-family ATPase protein, expressed |
| OS03G0584400 | AAA-family ATPase putative, expressed |
| OS03G0802500 | AAA-family ATPase putative, expressed |
| OS07G0192800 | AAA-family ATPase, putative, expressed |
| OS11G0244300 | AAA-type ATPase family protein, putative, expressed |

**Table S5.** ATPase related genes with similar expression levels in WT and *psd128* (*P ≥* 0.05)

| Gene Identifier | p-value | Description |
| --- | --- | --- |
| OS01G0605100 | NA | AAA-family ATPase, putative, expressed |
| OS03G0802600 | NA | ATPase, putative, expressed |
| OS05G0588900 | NA | AAA-family ATPase putative, expressed |
| OS06G0697500 | NA | AAA-type ATPase family protein, putative, expressed |
| OS06G0697600 | NA | ATPase, putative, expressed |
| OS07G0192000 | NA | ATPase, putative, expressed |
| OS07G0192700 | NA | ATPase , putative, expressed |
| OS07G0192800 | 0.18 | ATPase protein, putative, expressed |
| OS07G0517800 | 0.60 | AAA-type ATPase family protein, putative, expressed |
| OS10G0519300 | NA | AAA-type ATPase family protein, putative, expressed |
| OS12G0467700 | NA | ATPase 3, putative, expressed |
| OS12G0470700 | NA | AAA-type ATPase family protein, putative, expressed |
| OS12G0471100 | NA | ATPase 2, putative, expressed |
| OS12G0472300 | NA | ATPase 3, putative, expressed |
| OS12G0639400 | NA | AAA-type ATPase family protein, putative, expressed |
| OS12G0639500 | NA | ATPase 2, putative, expressed |

**Supplementary Dataset**

Supplementary Dataset 1. Differentially expressed genes (DEGs).

Supplementary Dataset 2. GO enrichment analysis of DEGs.

Supplementary Dataset 3. KEGG analysis.
